# Comparative Genomics of Mycobacterium avium Complex Reveals Signatures of Environment-Specific Adaptation and Community Acquisition

**DOI:** 10.1128/mSystems.01194-21

**Published:** 2021-10-19

**Authors:** Eric C. Keen, JooHee Choi, Meghan A. Wallace, Michelle Azar, Carlos R. Mejia-Chew, Shail B. Mehta, Thomas C. Bailey, Lindsay J. Caverly, Carey-Ann D. Burnham, Gautam Dantas

**Affiliations:** a The Edison Family Center for Genome Sciences and Systems Biology, Washington University School of Medicine in St. Louis, St. Louis, Missouri, USA; b Department of Pathology and Immunology, Washington University School of Medicine in St. Louis, St. Louis, Missouri, USA; c Department of Pediatrics, University of Michigan Medical School, Ann Arbor, Michigan, USA; d Department of Medicine, Washington University School of Medicine in St. Louis, St. Louis, Missouri, USA; e Department of Pediatrics, Washington University School of Medicine in St. Louis, St. Louis, Missouri, USA; f Department of Molecular Microbiology, Washington University School of Medicine in St. Louis, St. Louis, Missouri, USA; g Department of Biomedical Engineering, Washington University in St. Louis, St. Louis, Missouri, USA; University of California San Diego

**Keywords:** comparative genomics, genomic epidemiology, *Mycobacterium*, *Mycobacterium avium* complex, nontuberculous mycobacteria, whole-genome sequencing

## Abstract

Nontuberculous mycobacteria, including those in the Mycobacterium avium complex (MAC), constitute an increasingly urgent threat to global public health. Ubiquitous in soil and water worldwide, MAC members cause a diverse array of infections in humans and animals that are often multidrug resistant, intractable, and deadly. MAC lung disease is of particular concern and is now more prevalent than tuberculosis in many countries, including the United States. Although the clinical importance of these microorganisms continues to expand, our understanding of their genomic diversity is limited, hampering basic and translational studies alike. Here, we leveraged a unique collection of genomes to characterize MAC population structure, gene content, and within-host strain dynamics in unprecedented detail. We found that different MAC species encode distinct suites of biomedically relevant genes, including antibiotic resistance genes and virulence factors, which may influence their distinct clinical manifestations. We observed that M. avium isolates from different sources—human pulmonary infections, human disseminated infections, animals, and natural environments—are readily distinguished by their core and accessory genomes, by their patterns of horizontal gene transfer, and by numerous specific genes, including virulence factors. We identified highly similar MAC strains from distinct patients within and across two geographically distinct clinical cohorts, providing important insights into the reservoirs which seed community acquisition. We also discovered a novel MAC genomospecies in one of these cohorts. Collectively, our results provide key genomic context for these emerging pathogens and will facilitate future exploration of MAC ecology, evolution, and pathogenesis.

**IMPORTANCE** Members of the Mycobacterium avium complex (MAC), a group of mycobacteria encompassing M. avium and its closest relatives, are omnipresent in natural environments and emerging pathogens of humans and animals. MAC infections are difficult to treat, sometimes fatal, and increasingly common. Here, we used comparative genomics to illuminate key aspects of MAC biology. We found that different MAC species and M. avium isolates from different sources encode distinct suites of clinically relevant genes, including those for virulence and antibiotic resistance. We identified highly similar MAC strains in patients from different states and decades, suggesting community acquisition from dispersed and stable reservoirs, and we discovered a novel MAC species. Our work provides valuable insight into the genomic features underlying these versatile pathogens.

## INTRODUCTION

Nontuberculous mycobacteria (NTM), or mycobacterial species other than Mycobacterium tuberculosis complex species and Mycobacterium leprae, are ubiquitous in natural environments and important pathogens of humans and animals (1). Globally distributed in waters and soils, these diverse organisms cause equally diverse infections, from systemic mycobacteriosis in birds to gastrointestinal wasting in ruminants to soft tissue abscesses, chronic lymphadenitis, osteomyelitis, and disseminated disease in humans (2, 3). NTM are most notorious as pulmonary pathogens, and in patients with underlying lung conditions (e.g., cystic fibrosis or bronchiectasis), these infections are challenging to eradicate and sometimes fatal (4, 5). NTM infections are also increasing in frequency worldwide (6). For example, clinical NTM isolations in Ontario, Canada, nearly doubled from 1998 to 2010 (7), and in Taiwan, the incidence of NTM pulmonary disease increased more than 6-fold from 2000 to 2008 (8). Today, the prevalence of NTM disease is higher than that of tuberculosis in many countries, including the United States (9, 10). Thus, NTM represent an urgent and growing threat to global public health.

More than 160 NTM species have been described to date, but most NTM pulmonary disease is caused by Mycobacterium avium and nine closely related species, which collectively comprise the M. avium complex (MAC) (11). These 10 Mycobacterium species, M. avium, M. intracellulare, M. colombiense, M. arosiense, M. vulneris, M. bouchedurhonense, M. timonense, M. marseillense, M. paraintracellulare, and M. lepraemurium, are defined and differentiated genomically, and proposals for taxonomic rearrangement are ongoing (11, 12). However, despite the emergence of MAC and the utility of whole-genome sequencing (WGS) in characterizing clinically relevant pathogens, key aspects of MAC biology remain unresolved. Here, we leveraged comparative genomics and a unique cohort of isolates to explore three questions central to MAC pathogenesis, ecology, and evolution. First, we investigated whether the two most prominent MAC species, M. avium and M. intracellulare (13, 14), differ from each other and other complex members in their repertoires of biomedically significant genes, such as virulence factor (VF) genes and antibiotic resistance genes (ARGs). Second, we assessed whether MAC genomes display signatures of niche-specific adaptation, reflecting their versatility as free-living organisms and pathogens. Finally, we examined whether genomic epidemiology could provide new insights into MAC acquisition and transmission, as was recently described for another pathogenic NTM, Mycobacteroides abscessus complex (15).

To test these and related questions, we first profiled conserved genomic regions to establish population structures and phylogenetic relationships across MAC members and within M. avium. Next, we compared isolates’ accessory genomes to identify specific genes, including VF genes and ARGs, associated with particular MAC species and environmental niches. We then inspected MAC genomes for signatures of horizontal gene transfer (HGT) to infer how mobile genetic elements (MGEs) have shaped MAC genotypes and phenotypes. Finally, we utilized two unpublished clinical cohorts, accumulated from two hospitals over nearly a decade, to quantify intra- and interpatient MAC dynamics with strain-level resolution. This work represents the most comprehensive genomic characterization of the MAC to date and provides numerous testable hypotheses for further investigation *in vitro* and *in vivo*.

## RESULTS

### Species distribution of the MAC cohort.

**FIG 1:**
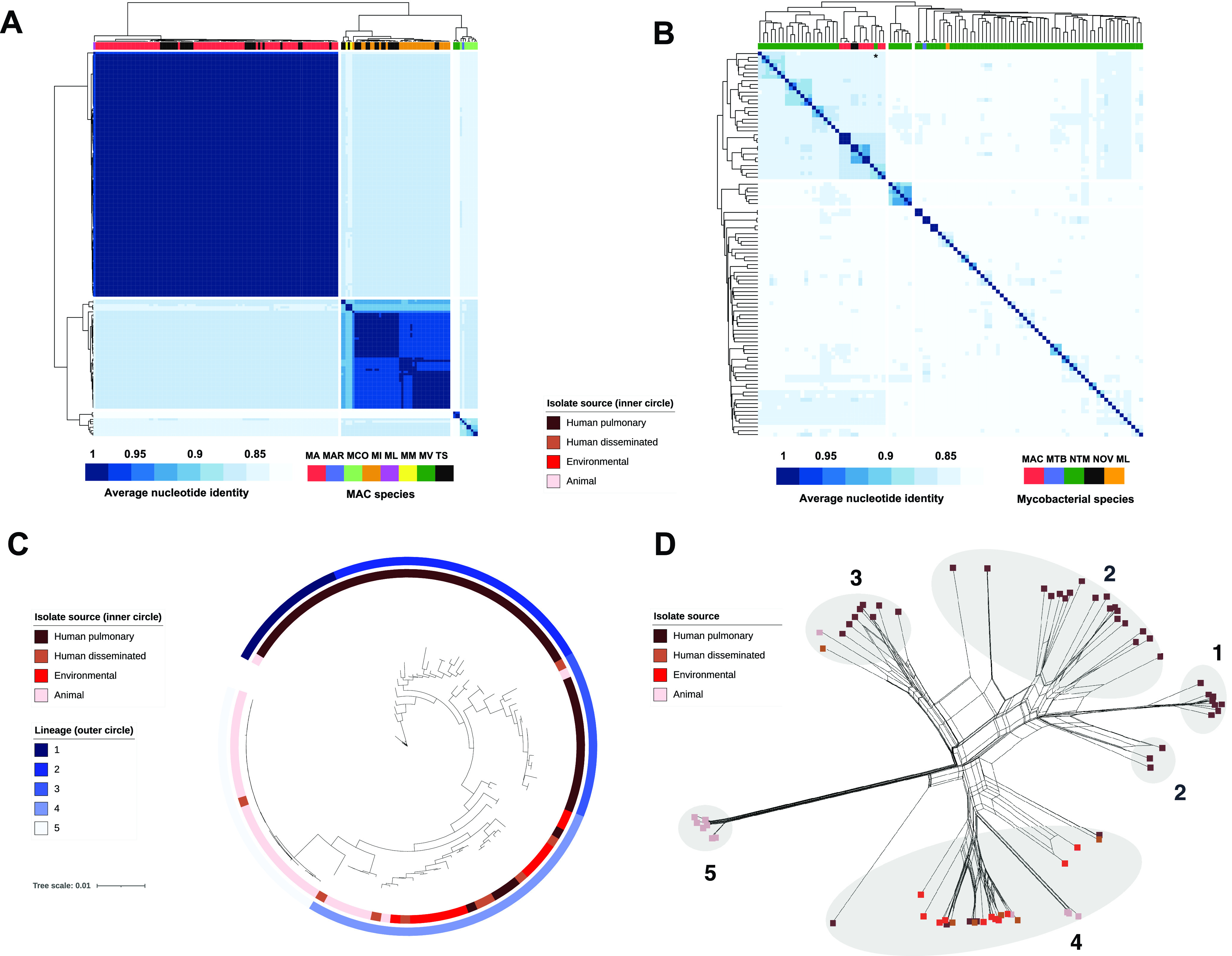
M. avium core genomes cluster by source of isolation. (A) Pairwise average nucleotide identity (ANI) matrix of all 170 MAC isolates in this study. MA, M. avium; MAR, *M. arosiense*; MCO, *M. colombiense*; MI, M. intracellulare; ML, M. leprae*murium*; MM, *M. marseillense*; MV, *M. vulneris*; TS, novel isolates from WUMAC and FLAC cohorts described in this study. (B) ANI matrix of all 96 available mycobacterial representative/reference strains and 2 WUMAC isolates with <95% ANI to any other isolate in panel A. MAC, M. avium complex; NTM, all other nontuberculous mycobacteria; MTB, M. tuberculosis; ML, M. leprae, NOV, WUMAC isolates which represent a putative novel MAC genomospecies. An asterisk denotes *M. mantenii*. (C) Core genome phylogeny of 109 M. avium isolates as determined by Roary and RAxML. The scale bar represents the number of substitutions per site. (D) Core genome phylogenetic network of 109 M. avium isolates as determined by SplitsTree4. Branch lengths represent uncorrected *P* values. Numbers represent lineages as shown in panel C. Overlapping nodes were not annotated with the isolate source.

Our MAC cohort contained 126 high-quality published genomes and an additional 44 newly sequenced genomes from two clinical cohorts: 27 genomes from 18 patients associated with Barnes-Jewish Hospital and Washington University School of Medicine in St. Louis (WUMAC isolates) and 17 genomes from 14 patients associated with the University of Michigan Medical School (FLAC isolates) (see Materials and Methods and Tables S1 and S2 in the supplemental material). All WUMAC and FLAC isolates were obtained from clinically significant pulmonary MAC infections. We first calculated pairwise average nucleotide identity (ANI), the genomic gold standard for defining microbial species (16), across all 170 isolates. The resulting ANI distribution revealed a cohort dominated by M. avium (109 genomes; 64%) and M. intracellulare (44 genomes; 26%) and containing five additional MAC species: M. vulneris, M. colombiense, M. marseillense, M. arosiense, and M. lepraemurium (Fig. 1A). Of our 44 newly sequenced isolates, 25 were identified by ANI as M. avium, 16 as M. intracellulare, and 1 as *M. marseillense*, closely mirroring matrix-assisted laser desorption ionization–time of flight (MALDI-TOF) mass spectrometry (MS) predictions for these isolates (Table S2).

10.1128/mSystems.01194-21.6 TABLE S1 One hundred seventy high-quality Mycobacterium avium complex genomes used in this study. Download Table S1, DOCX file, 0.03 MB. Copyright © 2021 Keen et al. 2021 Keen et al. https://creativecommons.org/licenses/by/4.0/ This content is distributed under the terms of the Creative Commons Attribution 4.0 International license.

10.1128/mSystems.01194-21.7 TABLE S2 Clinical metadata and species identification of WUMAC and FLAC isolates based on MALDI-TOF mass spectrometry and average nucleotide identity (ANI). All WUMAC and FLAC isolates were obtained from clinically significant MAC infections. Patient identity is the same as shown in Fig. 4. ND, not determined; MA, M. avium; MI, M. intracellulare; MM, *M. marseillense*; MN, novel MAC species. Age is at the time of first sampling. PHTN, pulmonary hypertension; GERD, gastroesophageal reflux disease; CF, cystic fibrosis; ABPA, allergic bronchopulmonary aspergillosis; COPD, chronic obstructive pulmonary disease; PLWHIV, person living with HIV; IBD, inflammatory bowel disease; CHF, congestive heart failure; CKD, chronic kidney disease; DM, diabetes mellitus; RA, rheumatoid arthritis; CFRD, cystic fibrosis-related diabetes. Download Table S2, DOCX file, 0.02 MB. Copyright © 2021 Keen et al. 2021 Keen et al. https://creativecommons.org/licenses/by/4.0/ This content is distributed under the terms of the Creative Commons Attribution 4.0 International license.

### Identification of novel MAC genomospecies.

Intriguingly, two additional isolates collected ∼26 months apart from the same WUMAC patient, WUMAC-027 and WUMAC-065, showed <93.8% ANI to all other 168 MAC genomes. A threshold of 95% ANI typically demarcates different species (16), implying that these isolates represent a distinct mycobacterial genomospecies. To confirm this finding, we downloaded representative or reference genomes for all available Mycobacterium and *Mycobacteroides* species (*n* = 96) and calculated pairwise ANIs for WUMAC-025 and WUMAC-067 (Fig. 1B). Despite the increased diversity of mycobacterial index genomes for comparison, the closest match for WUMAC-025 and WUMAC-067 was M. intracellulare ATCC 13950, with 92.9% ANI. We therefore suggest that isolates WUMAC-025 and WUMAC-067 are not closely related to a non-MAC species of mycobacteria but instead likely represent a novel genomospecies of the MAC. Our whole-genome ANI analysis, in contrast to previous comparisons of marker gene sequences (17), also supports the placement of Mycobacterium mantenii as an additional species within the MAC (Fig. 1B).

### M. avium core genomes cluster by isolate source.

Unlike both M. tuberculosis complex and numerous other NTM species, M. avium is relatively unique in its ability to cause life-threatening pulmonary and disseminated infection, infect other mammals and birds, and thrive in water, soil, and other natural environments. Our M. avium cohort included isolates from human pulmonary and disseminated infections (56 and 9 isolates, respectively), animals (31 isolates), and soil (13 isolates). Given its prevalence within the MAC (13, 14) and its ability to occupy diverse niches, we selected M. avium for further genomic characterization. We annotated all 109 M. avium genomes and generated a core genome alignment from 3,425 core genes, which we then used to construct a maximum likelihood phylogenetic tree (Fig. 1C). The resulting phylogeny yielded five distinct lineages of isolates (Fig. S1A). As expected, lineages differed by subspecies, with all M. avium subsp. *paratuberculosis* (MAP) isolates forming their own discrete lineage (lineage 5). Intriguingly, we also observed strong clustering by M. avium isolate source (Fig. 1C; Fig. S1A). Lineages 1 to 3 comprised human pulmonary isolates almost exclusively (51 of 54 isolates), while lineage 5 was dominated by genomes of animal origin (23 of 24 isolates). In contrast, lineage 4 displayed substantial heterogeneity and included all 13 environmental isolates, 6 animal isolates, and 7 and 5 isolates from human disseminated and pulmonary infections, respectively. These human pulmonary isolates from lineage 4 (*n* = 5) bore numerous genes which were significantly less abundant in human pulmonary isolates from the more homogenous lineages 1 through 3 (*n* = 51) (Table S3). Lineage composition was significantly nonrandom (*P* < 0.0001; chi-square test), suggesting that in M. avium, genomic diversity accompanies habitat diversity and that specific genotypes are associated with specific niches.

10.1128/mSystems.01194-21.1 FIG S1 Different M. avium lineages are dominated by isolates from different sources, and M. avium has an open pangenome. (A) Lineage profiling from hierBAPS/fastGEAR analysis of M. avium core genomes (*n* = 109). Lineage 5 is exclusively comprised of M. avium subsp. *paratuberculosis* isolates. Lineage clusters are significantly nonrandom (*P* < 0.0001; chi-square test). (B) Total genes (dashed line) and core genes (solid line) in 109 M. avium isolates. Additional genes are identified upon the inclusion of additional genomes (*n* = 25 from this study). Download FIG S1, PDF file, 0.03 MB. Copyright © 2021 Keen et al. 2021 Keen et al. https://creativecommons.org/licenses/by/4.0/ This content is distributed under the terms of the Creative Commons Attribution 4.0 International license.

10.1128/mSystems.01194-21.8 TABLE S3 Gene annotations differentially abundant between human pulmonary isolates from heterogenous lineage 4 (*n* = 5) and all other sources (*n* = 51) as determined by Scoary (*P* < 10^−5^). Positive and negative fold changes refer to genes that were more abundant in human pulmonary isolates from lineage 4 and other lineages, respectively. Download Table S3, DOCX file, 0.03 MB. Copyright © 2021 Keen et al. 2021 Keen et al. https://creativecommons.org/licenses/by/4.0/ This content is distributed under the terms of the Creative Commons Attribution 4.0 International license.

Phylogenetic networks complement traditional phylogenetic trees by incorporating intertree variation and by better accounting for reticulate evolution, including horizontal gene transfer (HGT), hybridization, and duplication (18). Therefore, we conducted neighbor-net analysis on all 109 M. avium core genomes (Fig. 1D). Consistent with previous reports (19), we observed diminished genomic diversity within MAP relative to that of other M. avium subspecies. Critically, the resulting phylogenetic network closely resembled the genomic relationships depicted in Fig. 1C, suggesting a lack of significant ancestral recombination between isolate sources and lineages, consistent with largely independent evolutionary histories.

### M. avium pangenomes cluster by isolate source and encode niche-specific genes.

**FIG 2:**
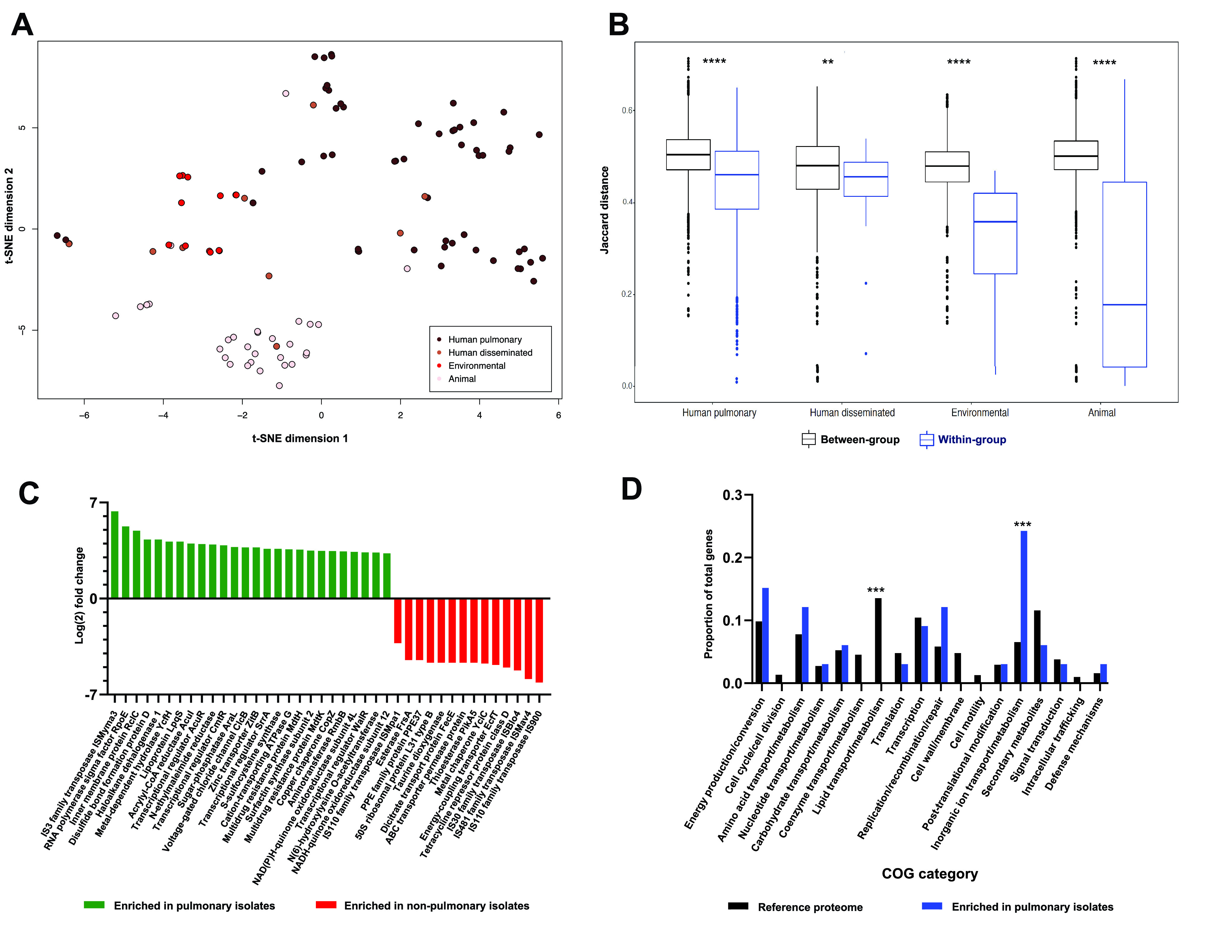
M. avium pangenomes cluster by source of isolation. (A) Pangenome ordination of 109 M. avium isolates by t-distributed stochastic neighbor embedding (t-SNE). (B) Between- and within-group Jaccard dissimilarity of M. avium isolate sources, comprising 109 total isolates. ****, *P* < 0.0001; **, *P* < 0.01 (Kruskal-Wallis test with the Benjamini-Hochberg correction). (C) Genes differentially abundant in pulmonary (*n* = 56; green) and nonpulmonary (*n* = 53; red) M. avium isolates as identified by Scoary. All genes with substantial (log_2_ fold change > 3) and significant (*P* < 0.05; Fisher’s exact test with the Benjamini-Hochberg correction) enrichment are shown. (D) COG profiles of enriched genes in panel C. Categories of general or unidentified hits or those with no hits are not shown. The reference proteome represents the COG distribution of proteins in the representative M. avium strain OCU464. ***, *P* < 0.001 (Fisher’s exact test with the Benjamini-Hochberg correction).

We next considered the possibility that M. avium pangenomes, like core genomes, cluster by isolate source. Consistent with previous reports (20), we observed an open pangenome in M. avium (Fig. S1B) despite the addition of 25 newly sequenced genomes from this study. The M. avium accessory genome was considerably larger than the core genome (10,883 and 3,425 genes, respectively), implying that numerous gene functions may be differentially abundant across M. avium isolates and lineages. Indeed, upon unsupervised ordination of all isolates’ pangenome contents, we again observed strong clustering by isolate source (Fig. 2A). When grouped by isolate source, M. avium pangenomes also displayed significantly greater within-source similarity (Jaccard index) than between-source similarity (*P* < 0.01; Kruskal-Wallis test with the Benjamini-Hochberg correction) (Fig. 2B). These results collectively demonstrate that in M. avium, both core genomes and pangenomes differ by environment of origin and, additionally, imply that M. avium bears accessory genes which facilitate niche-specific adaptation.

Given these associations between genomes and habitats, we hypothesized that the presence or absence of specific accessory genes might distinguish human pulmonary isolates from human disseminated, animal, and environmental isolates. We therefore quantitatively profiled the pangenomic contents of our 56 pulmonary and 53 nonpulmonary M. avium genomes. We identified numerous genes which were significantly enriched in human pulmonary genomes relative to their representation in isolates from other sources (*P* < 0.05; Fisher’s exact test with the Benjamini-Hochberg correction) (Fig. 2C; Table S4). These gene products included a lipoprotein associated with virulence in M. tuberculosis (LpqS) (21), regulators of virulence and immune evasion in other Gram-positive pathogens (WalR and SrrA) (22, 23), multidrug resistance proteins (MdtH and MdtK) (24), components of metal transport machinery (CopZ and ZitB) (25, 26), and a membrane protein associated with resistance to reactive chlorine species (RclC) (27). Intriguingly, three separate subunits of bacterial NAD(P)H-quinone oxidoreductase, an enzyme thought to assist the intracellular survival of M. tuberculosis via detoxification of host-derived metabolites and/or energy generation under hypoxic conditions (28), were also significantly enriched in human pulmonary isolates. These and other genes (Table S4) represent plausible candidates for *in vitro* and *in vivo* characterization in the context of M. avium pulmonary pathogenesis.

10.1128/mSystems.01194-21.9 TABLE S4 Gene annotations differentially abundant between human pulmonary isolates (*n* = 56) and all other sources (*n* = 53) as determined by Scoary (*P* < 10^−5^; Fisher’s exact test with the Benjamini-Hochberg correction). Positive and negative fold changes refer to genes more abundant in pulmonary and nonpulmonary genomes, respectively. Download Table S4, DOCX file, 0.03 MB. Copyright © 2021 Keen et al. 2021 Keen et al. https://creativecommons.org/licenses/by/4.0/ This content is distributed under the terms of the Creative Commons Attribution 4.0 International license.

To gain further insight into gene functions enriched in pulmonary isolates, we binned differentially abundant genes into clusters of orthologous protein groups (COGs). Although this enriched gene set and the proteome of the representative M. avium strain OCU464 had generally similar COG distributions, proteins putatively involved in lipid transport and metabolism (COG category I) were entirely absent among differentially abundant hits despite being well represented in the M. avium OCU464 proteome (13.5% of annotations) (Fig. 2D). Conversely, annotations for inorganic ion transport and metabolism (COG category P) were significantly overrepresented in Scoary hits (22.9% of annotations) relative to those of the M. avium OCU464 proteome as a whole (5.2%) (*P* < 0.001; Fisher’s exact test with the Benjamini-Hochberg correction) (Fig. 2D). These results indicate a nonrandom pattern of gene enrichment and are consistent with numerous reports that metal acquisition is essential for intracellular persistence and virulence in mycobacterial lung infections (29, 30).

### Horizontal gene transfer varies by MAC species and M. avium isolate source.

**FIG 3:**
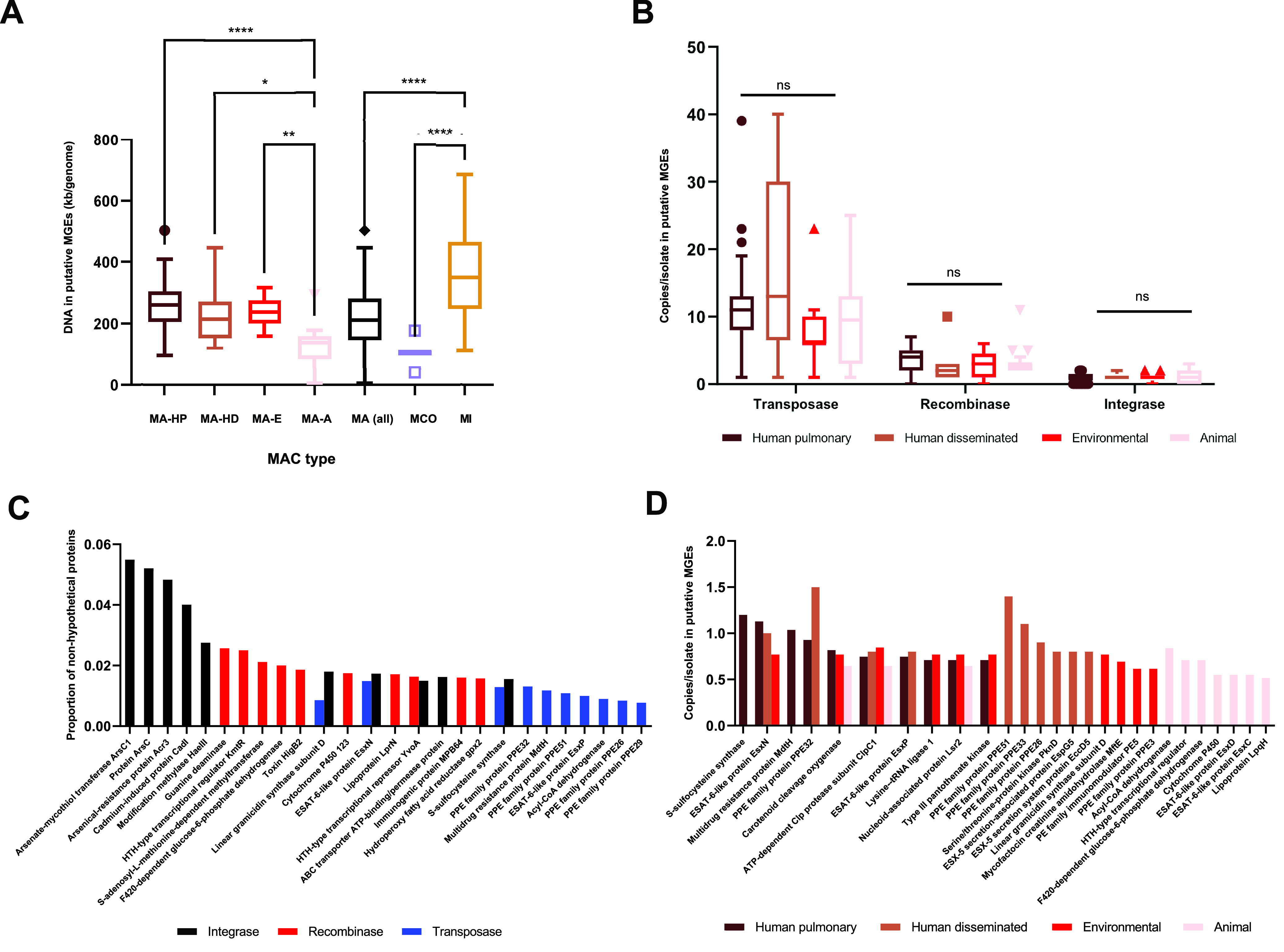
Foreign DNA signatures vary by MAC species and M. avium isolate source. (A) Total numbers of kilobases of putative foreign DNA in MAC genomes as identified by IslandPath-DIMOB. MA-HP, human pulmonary M. avium isolates; MA-HD, human disseminated M. avium isolates; MA-E, environmental M. avium isolates; MA-A, animal M. avium isolates; MI, M. intracellulare; MCO, *M. colombiense*. ****, *P* < 0.0001; **, *P* < 0.01; *, *P* < 0.05 (one-way ANOVA with Tukey’s *post hoc* correction). (B) Total numbers of copies of mobility elements within foreign DNA in M. avium, binned by isolate source. ns, not significant (one-way ANOVA with Tukey’s *post hoc* correction for each mobility element). (C) Top 10 most abundant genes within each mobility element in foreign DNA in M. avium. Mobility and hypothetical proteins are not shown. (D) Top 10 most abundant genes within foreign DNA for each M. avium isolate source. Mobility and hypothetical proteins are not shown.

Given the presence of several transposases as top differentially abundant genes (Fig. 2C) in our data set, as well as the variable importance of HGT in the evolution of various mycobacteria (31–34), we next profiled HGT dynamics in MAC species. We observed clear differences in HGT burden across MAC species, with M. intracellulare containing significantly more foreign DNA than M. avium and *M. colombiense* (*P* < 0.0001; one-way analysis of variance [ANOVA] with Tukey’s *post hoc* correction) (Fig. 3A). In human and environmental M. avium isolates, foreign DNA represented a fraction (4 to 5%) of the genome comparable to the 4.5% reported for M. tuberculosis (35, 36). Surprisingly, however, animal isolates encoded significantly less foreign DNA than all other M. avium isolate sources (*P* < 0.05; one-way ANOVA with Tukey’s *post hoc* correction) (Fig. 3A). This reduced evidence of HGT in animal isolates, particularly but not exclusively in M. avium subsp. *paratuberculosis*, is consistent with the tight clustering of lineage 5 in Fig. 1D and with previous reports (19), indicating potential differences in the ecologies and/or genome biologies of animal isolates.

We next characterized these regions of foreign DNA on the basis of their mobility genes. The vast majority of foreign regions were associated with putative transposases, recombinases, or bacteriophage-derived integrases. Although the genomic abundance of these mobilization elements did not vary significantly by isolate source (*P* > 0.05; one-way ANOVA with Tukey’s *post hoc* correction) (Fig. 3B), we found that different elements were enriched for distinct genes (Fig. 3C). For example, of the 10 nonhypothetical proteins most commonly associated with transposases, the most abundant MGE class in our genomes, only 3 were among the top 10 associated with recombinases or integrases. We observed that PPE proteins, an abundant and enigmatic mycobacterial protein family linked to immune evasion (37) and nutrient transport (38), were commonly colocalized with transposases but rarely with integrases or recombinases (Fig. 3C). PPE genes have previously been associated with insertion sequences in M. tuberculosis (39) but never, to our knowledge, in M. avium. Additionally, a cluster of metal-related genes, including the arsenate reductase gene *arsC* and cadmium-associated protein gene *cadI*, were strongly associated with integrases, consistent with the previous discovery of *arsC* in diverse temperate phage genomes (40). These results suggest that different MGEs encode overlapping but largely distinct gene repertoires in M. avium.

We further speculated that MGE-borne genes, like genomic content as a whole (Fig. 2), might also vary by source of isolation in M. avium. We therefore binned putative foreign regions by isolate source and compared gene annotations across bins. As predicted, we observed different MGE-encoded gene profiles in different isolate sources (Fig. 3D), albeit with greater overlap across groups than when binned by MGE type (Fig. 3C). Consistent with the paradigm of genome islands being VF hot spots, including in M. tuberculosis (35), we identified several canonical mycobacterial VF genes within these regions, including PPE and ESAT-6-like proteins (41). Several other top hits, including *S*-sulfocysteine synthase (*cysK2*) and carotenoid cleavage oxygenase (*Rv0654*), have putative but largely unexplored roles in mycobacterial infection (42, 43), and our findings support their further investigation. More broadly, our results suggest that HGT profiles vary by species, habitat, and MGE type across the MAC, with potentially wide-ranging implications for phenotypic variation and pathogenesis.

### Virulence factors vary by MAC species and M. avium isolate source.

Like other bacterial pathogens, NTM encode a variety of VFs which collectively enable immune evasion and host antagonism (41, 44). Given the genomic distinctions described in Fig. 1 and 3, we hypothesized that virulence gene profiles would also differ between various MAC species and isolate sources. We therefore generated a custom BLAST database of 9,114 protein sequences from three virulence factor databases (45–47) and queried proteomes from all 170 MAC isolates against this database. With thresholds of 80% sequence identity and coverage, we identified 244 VF homologs present in at least one MAC isolate. Of these, 47 were significantly differentially abundant between M. avium and all other MAC species (*P* < 0.05; Fisher’s exact test with the Benjamini-Hochberg correction) (Table S5), and we observed clear clustering both by MAC species and by M. avium isolate source (Fig. S2). Within M. avium, 13 VFs were significantly differentially abundant between human pulmonary and nonpulmonary isolates (*P* < 0.05; Fisher’s exact test with the Benjamini-Hochberg correction) (Fig. S3). Consistent with the results shown in Fig. 2D, several of these proteins, including the siderophore transporter IrtA (48) and the siderophore acyltransferase MbtK (49), are involved in metal acquisition and transport in mycobacteria. Intriguingly, a protein uniquely present in nonpulmonary M. avium isolates, the heparin-binding hemagglutinin HbhA, is also required for extrapulmonary dissemination in M. tuberculosis (50). In a mouse model of tuberculosis, *hbhA* mutants were severely deficient (200-fold) in spleen colonization, but not in lung colonization, following intranasal inoculation (50). Our results support a similar putative function for HbhA in M. avium and, more broadly, illuminate additional genes which potentially underlie different forms of infection by this versatile pathogen.

10.1128/mSystems.01194-21.2 FIG S2 MAC species and M. avium isolate sources differ in their encoded virulence factors. Hierarchical clustering of 244 mycobacterial virulence factors present in at least one MAC genome. MA-HP, human pulmonary M. avium isolates; MA-HD, human disseminated M. avium isolates; MA-E, environmental M. avium isolates; MA-A, animal M. avium isolates; MAR, *M. arosiense*; MCO, *M. colombiense*; MI, M. intracellulare; ML, M. leprae*murium*; MM, *M. marseillense*; MV, *M. vulneris*. Download FIG S2, PDF file, 0.2 MB. Copyright © 2021 Keen et al. 2021 Keen et al. https://creativecommons.org/licenses/by/4.0/ This content is distributed under the terms of the Creative Commons Attribution 4.0 International license.

10.1128/mSystems.01194-21.3 FIG S3 Specific virulence factors distinguish human pulmonary M. avium isolates. All virulence factors from Fig. S3 which significantly differ (*P* < 0.05 by Fisher’s exact test with the Benjamini-Hochberg correction; *n* = 13) between human pulmonary (*n* = 56) and all other (*n* = 53) M. avium isolates. Download FIG S3, PDF file, 0.03 MB. Copyright © 2021 Keen et al. 2021 Keen et al. https://creativecommons.org/licenses/by/4.0/ This content is distributed under the terms of the Creative Commons Attribution 4.0 International license.

10.1128/mSystems.01194-21.10 TABLE S5 Virulence factors differentially abundant between M. avium isolates (*n* = 109) and all other MAC species (*n* = 61). All significant genes (*P* < 0.05; Fisher’s exact test with the Benjamini-Hochberg correction) are shown. Download Table S5, DOCX file, 0.02 MB. Copyright © 2021 Keen et al. 2021 Keen et al. https://creativecommons.org/licenses/by/4.0/ This content is distributed under the terms of the Creative Commons Attribution 4.0 International license.

### *M. vulneris* encodes unique antibiotic resistance genes.

Both pulmonary and disseminated MAC infections are notoriously refractory to antibiotic treatment (5, 6). Along with having innate resistance to entire classes of antibiotics, NTM can acquire antibiotic resistance via mutation and, less commonly, through HGT (51–53). These factors usually necessitate the adoption of lengthy multidrug treatment regimens involving some combination of macrolides, aminoglycosides, rifampin, and ethambutol (54, 55). We therefore used two complementary approaches to profile antibiotic resistance in MAC genomes. First, we manually inspected each 16S (*rrs*) and 23S (*rrl*) rRNA gene sequence for point mutations in positions 1406 to 1408 and 2058 to 2059, which confer resistance to aminoglycosides and macrolides, respectively (56, 57). We found these mutations to be relatively uncommon overall (in 5 and 7 of 170 total isolates, respectively), most common in M. intracellulare (in 3 and 4 of 44 isolates, respectively), and present only in human pulmonary isolates (Fig. S4). Next, we queried the MEGARes 2.0 resistance gene database (58) to identify ARGs more comprehensively. Nearly all MAC isolates encoded the same three intrinsic ARGs: the genes for an RNA polymerase binding protein associated with rifampin tolerance (*rbpA*) (59), an efflux pump (*efpA*) (60), and an efflux regulator (*mtrA*) (61) (Fig. S4). We also detected several additional ARGs, including the class A β-lactamase *blaF*, in all three isolates of *M. vulneris*, a recently described MAC species (62). To our knowledge, this is the first report of *blaF* in a MAC genome (63) and the first comprehensive characterization of ARGs across all MAC species.

10.1128/mSystems.01194-21.4 FIG S4 Antibiotic resistance genes and mutations are infrequent but variable in MAC genomes. Hierarchical clustering of key antibiotic resistance genes (*n* = 9) and point mutations (*n* = 2) present in at least one MAC genome. MA-HP, human pulmonary M. avium isolates; MA-HD, human disseminated M. avium isolates; MA-E, environmental M. avium isolates; MA-A, animal M. avium isolates; MAR, *M. arosiense*; MCO, *M. colombiense*; MI, M. intracellulare; ML, M. leprae*murium*; MM, *M. marseillense*; MV, *M. vulneris*. Download FIG S4, PDF file, 0.1 MB. Copyright © 2021 Keen et al. 2021 Keen et al. https://creativecommons.org/licenses/by/4.0/ This content is distributed under the terms of the Creative Commons Attribution 4.0 International license.

### Long-term and geographically dispersed reservoirs seed community acquisition of MAC organisms.

**FIG 4:**
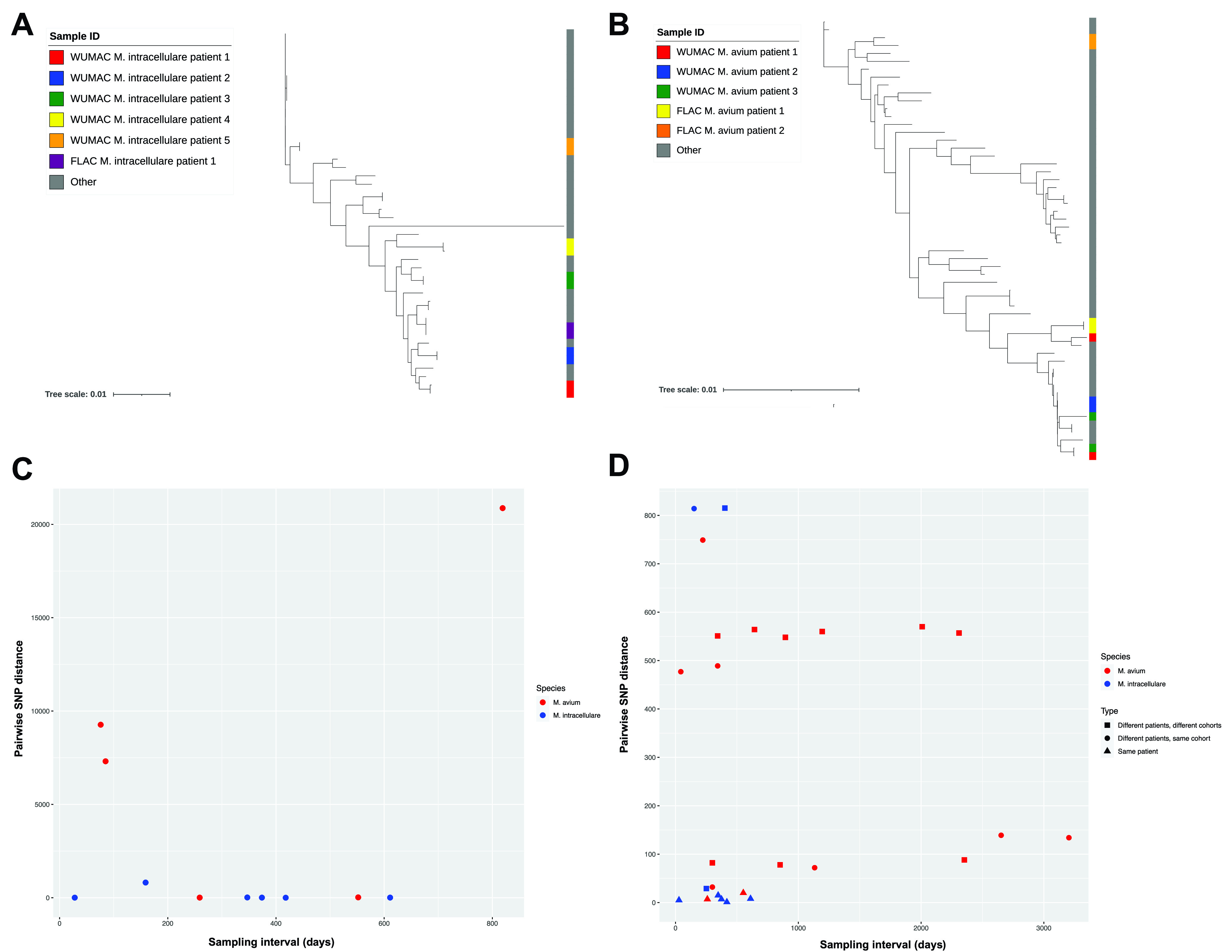
Similar MAC strains in distinct patients and cohorts. (A) Core genome phylogeny of all WUMAC and FLAC M. intracellulare isolates (*n* = 16) and all other M. intracellulare isolates (*n* = 28). Unpaired isolates are from patients for whom only a single isolate was available. The scale bar represents the number of substitutions per site. (B) Core genome phylogeny of all WUMAC and FLAC M. avium isolates (*n* = 25) and all other M. avium human pulmonary isolates (*n* = 31). Unpaired isolates are from patients for whom only a single isolate was available. The scale bar represents the number of substitutions per site. (C) Pairwise single-nucleotide polymorphism (SNP) distances for all intrapatient isolate pairs from WUMAC/FLAC M. avium (*n* = 5) and M. intracellulare (*n* = 6) patients. The sampling interval represents the number of days between isolate collections. (D) All intrapatient and interpatient pairwise core genome SNP distances that were <1,000 for all possible WUMAC/FLAC M. avium and M. intracellulare isolate pairs. Pairwise SNP distances of >1,000 are not shown. The sampling interval represents the number of days between isolate collections.

An important aspect of this work, and one which distinguishes it from other recent MAC comparative genomics studies (20, 64, 65), is our collection of longitudinal isolates from individual patients from multiple cohorts (Table S2). Our WUMAC and FLAC cohorts collectively contained pairs of pulmonary isolates from five patients with M. avium and six patients with M. intracellulare, separated by an average of 339 ± 244 days, which we leveraged to investigate within-host strain dynamics. We first sought to characterize the diversity of these paired isolates within their broader genomic context by generating core genome phylogenies of all M. avium pulmonary isolates (*n* = 56; *n* = 25 from this study) and all M. intracellulare isolates (*n* = 44; *n* = 16 from this study). Our WUMAC/FLAC isolates spanned the genomic breadth of both species, but while all M. intracellulare isolate pairs aligned together (Fig. 4A), paired isolates from WUMAC M. avium patients 1 and 3 were surprisingly diverse (Fig. 4B).

To quantify these dynamics with greater genomic resolution, we calculated pairwise single-nucleotide polymorphism (SNP) distances for all intrapatient M. avium and M. intracellulare isolate pairs in our WUMAC and FLAC cohorts. We observed a substantial gradient of SNP distances, with six highly similar isolate pairs (two M. avium and four M. intracellulare isolate pairs; SNP distance, ≤20) and five pairs (three M. avium and two M. intracellulare isolate pairs) comprising more distantly related strains (SNP distance, >400) (Fig. 4C). Because we did not sequence metagenomic sweeps or multiple colonies per sample, we cannot exclude the possibility of monoclonal infection followed, in some patients, by strain replacement. However, these results are more parsimonious with long-standing evidence of polyclonal and even multispecies mycobacterial lung disease in NTM patients (66–69).

Next, we examined pairwise core genome SNP distances for all M. avium and M. intracellulare isolates, regardless of patient, from our WUMAC and FLAC cohorts. Consistent with the results in Fig. 4A and B, we observed clear discrepancies by species: while 5 of the 8 most related M. intracellulare pairwise comparisons were from the same patients, 17 of the 19 closest M. avium pairings were from different patients (Fig. 4D). Importantly, many of these closely related interpatient isolates were also obtained across considerable time and space. For example, M. avium isolates WUMAC-026 and WUMAC-062 differed by <75 SNPs but were collected from discrete patients in January 2013 and March 2016, respectively. M. avium isolates WUMAC-20 and WUMAC-35 (SNP distance of 134) were collected from a 43-year-old patient living with cystic fibrosis and a 71-year-old patient living with rheumatoid arthritis, respectively, more than 8 years apart. Perhaps most remarkably, M. intracellulare isolates WUMAC-033 and FLAC0181 differed by just 24 SNPs despite being collected in different years from different hospitals more than 400 miles apart. Indeed, of the 26 isolate combinations with pairwise core SNP distances of <1,000, just 7 (27%) represented multiple isolates from the same patient (Fig. 4D).

Finally, we considered the possibility that despite possessing congruent core genomes (Fig. 4), some pairs of interpatient and intercohort isolates might encode divergent accessory genomes, thereby complicating assumptions of genomic relatedness. We therefore aligned reads from all WUMAC/FLAC M. avium isolates (*n* = 25) and M. intracellulare isolates (*n* = 16) to their respective representative NCBI strains (M. avium OCU464 and M. intracellulare ATCC 13950, respectively) and generated whole-genome SNP alignments. Critically, the resulting SNP distance distributions (Fig. S5) closely mirrored those from core genome alignments (Fig. 4D), further suggesting that comparable MAC isolates can be separated by considerable space and time. The striking similarity of isolates from such disparate sources raises intriguing questions about MAC reservoirs and acquisition in these cohorts and beyond, which we discuss below.

10.1128/mSystems.01194-21.5 FIG S5 Whole-genome SNP distance distributions for MAC isolates from WUMAC and FLAC cohorts. All intrapatient and interpatient pairwise whole-genome SNP distances of <1,000 for all possible WUMAC/FLAC M. avium and M. intracellulare isolate pairs. Pairwise SNP distances of >1,000 are not shown. The sampling interval represents the number of days between isolate collections. Download FIG S5, PDF file, 0.02 MB. Copyright © 2021 Keen et al. 2021 Keen et al. https://creativecommons.org/licenses/by/4.0/ This content is distributed under the terms of the Creative Commons Attribution 4.0 International license.

## DISCUSSION

The M. avium complex is an increasing threat to public health. The vast majority of NTM lung disease in the United States is caused by MAC strains, with annual prevalence nearly doubling between 2008 and 2015 (70). MAC infections are often chronic, multidrug resistant, and fatal, with an all-cause 5-year mortality of ∼25% for infected patients (4, 6). Several MAC subspecies, most notably MAP, are also prominent animal pathogens and cost hundreds of millions of dollars annually in culls and lost productivity (71, 72). Despite this obvious significance, however, MAC genomics remains understudied. Fewer than 300 MAC genomes, many of which are of low quality (high sequence heterogeneity or contamination and/or low completeness), have been deposited in GenBank, compared to >1,800 for the M. abscessus complex and >6,500 for the M. tuberculosis complex. In this work, we contribute an additional 44 high-quality MAC genomes, including two from a putative novel MAC genomospecies, and provide important insight into the comparative genomics of these emerging pathogens.

Despite substantial genotypic and phenotypic similarity, different MAC species vary in important ways. For instance, the complex’s two most medically significant species, M. avium and M. intracellulare, differ both by geographic distribution (55) and by clinical outcomes (13, 14). Here, we extend and contextualize these observations by identifying differences in three key elements of MAC genomes, namely, VF genes, ARGs, and regions of foreign DNA (Fig. S2 and S4; Fig. 3). These differences were most pronounced for *M. vulneris*, a recently discovered and heretofore genomically undescribed MAC species (62), which harbored more ARGs but fewer virulence genes than its relatives. We note that 3 of the 10 MAC species represented in the NCBI, *M. bouchedurhonense*, *M. timonense*, and *M. paraintracellulare*, were not included in this study because no high-quality genomes were available, while another 3 species (*M. arosiense*, *M. marseillense*, and M. leprae*murium*) were represented by two or fewer genomes. Our discovery of an additional novel MAC genomospecies (Fig. 1B) further illustrates that many more isolates, from many more species, will be required to fully capture the genomic intricacies of the microorganisms in this complex.

We focused deeper comparative analyses on M. avium because of its clinical importance, its preponderance within the MAC, and its diversity of occupied habitats. A central finding of this work is that M. avium isolates from different sources—human pulmonary and disseminated infections, animals, and free-standing environments—are genomically distinct by multiple measures of comparison. These parameters include core genomes (Fig. 1C and D; Fig. S1B), accessory genomes (Fig. 2A to B), and gene profiles (Fig. 2C and D), including for VF genes (Fig. S3) and mobile genetic elements (Fig. 3). The observed patterns cannot be explained simply by the presence of different M. avium subspecies in different environments, since pulmonary and nonpulmonary isolates of M. avium subsp. *hominissuis* were well represented in our cohort. Although M. avium genomes have previously been shown to differ by source, most prior work has involved variable number tandem repeat fingerprinting (73–77), which provides limited genomic resolution and minimal biological insight compared to whole-genome sequencing. Of the few studies to employ WGS (20, 64, 65), ours is the largest to date and the only one to capture the diversity of niches that M. avium may occupy. By leveraging WGS, we also identified numerous specific genes, many of which are potentially clinically significant, which differentiate human pulmonary isolates from others (Fig. 2C; Table S4). While these findings would be strengthened by a broader collection of genomes (most human isolates were from North America and Asia, while environmental isolates were predominantly European), this work provides novel insights into M. avium biology and represents a rich substrate for future investigation *in vitro* and *in vivo*. For instance, our study implicates multiple individual genes, including *hbhA*, *lpqS*, and *cysK2*, as being particularly important for pulmonary MAC infection, a hypothesis which could be verified via gene knockouts and experimental infections of cells or animals. More broadly, comparative dual RNA sequencing (RNA-seq) profiling (i.e., host-pathogen transcriptomics in parallel) (78) upon infection by representative clinical and environmental isolates could shed valuable light on the mechanisms which underlie and restrict MAC disease.

Along with extensively analyzing publicly available genomes, our study incorporated longitudinal samples from two unpublished clinical cohorts, which we used to compare intra- and interpatient MAC dynamics. A limitation of this work is that we were restricted by upstream clinical collection procedures to a single colony per patient sample. Since NTM patients can be coinfected by multiple mycobacterial strains or even multiple species (66–69), such sampling is insufficient to accurately recapitulate microbial diversity. Thus, our finding of higher intrapatient diversity for M. avium than for M. intracellulare (Fig. 4C) should be interpreted with considerable caution, while the presence of highly related isolates in highly dissimilar patients (Fig. 4D) may be the rule rather than the exception. As was recently described (69), future studies may employ deep metagenomic sequencing and analysis of metagenome-assembled genomes, rather than selecting individual colonies, in order to fully capture mycobacterial diversity in samples of interest. These data sets will be instrumental in addressing key outstanding questions, including whether MAC mutation rates differ by species and host features (79), which genes are most frequently mutated during chronic infection, and how horizontal gene transfer shapes features of polymicrobial lung communities.

The striking similarity that we observed between clinical isolates from unrelated WUMAC/FLAC patients is particularly intriguing in light of MAC transmission dynamics. Unlike M. tuberculosis infections, MAC infections are typically thought to arise from environmental exposure, not human-to-human transmission (1–3). Multiple fingerprinting studies have demonstrated high genomic similarity between paired clinical and environmental isolates from the same household (80–83), but until recently, this paradigm had not been supported by WGS data (84). In pioneering work, Lande et al. described matched respiratory and built environment isolates differing by <100 SNPs and implicated municipal water supplies as likely M. avium reservoirs (84). Since this study encompassed a very narrow patient cohort (adult women living <20 miles apart in Pennsylvania, USA), however, its generalizability was unknown. By demonstrating genomic similarities between isolates collected up to 8 years apart from patients without direct contact (Fig. 4D), our data provide important support for this model and imply long-term colonization of community-accessible reservoirs by multiple M. avium strains. Moreover, since some of our most closely related isolates were collected from separate medical centers in different states, our results also suggest that highly similar MAC strains are not only temporally stable within reservoirs but also geographically dispersed across reservoirs. Our work thus provides valuable insights into MAC acquisition dynamics and may facilitate interventions to slow the emergence of these versatile pathogens.

## MATERIALS AND METHODS

### Bacterial culturing and MALDI-TOF mass spectrometry.

WUMAC isolates were recovered from clinical specimens submitted to the Barnes-Jewish Hospital microbiology laboratory as part of routine clinical care between 2006 and 2019. All isolates were recovered from respiratory specimens, including sputum, tracheal aspirates, bronchial wash fluids, and bronchial alveolar lavage fluids, and were stored at −80°C prior to analysis. Isolates were cultured from freezer stocks onto Middlebrook 7H11 agar (Hardy Diagnostics, Santa Maria, CA) and incubated at 35°C in air. Following sufficient growth, the identity of each isolate was confirmed using Vitek MALDI-TOF MS with Knowledge Base 3.0 (bioMérieux, Durham, NC, USA). Briefly, each isolate was picked using a sterile loop, spotted onto a target, and overlaid with 1 μl of formic acid. Once dry, 1 μl of the matrix was overlaid and allowed to dry. Isolates that could not be identified by Vitek MS were analyzed using the MALDI Biotyper with the MBT Mycobacteria module (Bruker, Billerica, MA, USA) using the same spotting procedure. Outgrowths positively identified as MAC (M. avium, M. avium-M. intracellulare, or *M. chimaera-*M. intracellulare) by either method were collected by swabbing the growth on agar plates and eluting it into molecular-grade water for storage at −80°C prior to extraction. In total, 27 WUMAC isolates were obtained from clinical samples from 18 patients.

FLAC isolates were obtained from clinical specimens associated with the University of Michigan Medical School as previously described (85). In total, 17 FLAC isolates were obtained from clinical samples from 14 patients.

### DNA extraction, sequencing, and assembly.

WUMAC isolate suspensions were thawed and mechanically lysed for 2 min (Mini-Beadbeater-24; BioSpec, Bartlesville, OK, USA), followed by DNA extraction (QIAamp BiOstic bacteremia DNA kit; Qiagen, Germantown, MD, USA) and quantification (Qubit HS; Thermo Fisher Scientific; Waltham, MA, USA) according to manufacturers’ protocols. Genomic DNA (0.5 ng) was used as the input in the preparation of sequencing libraries (Nextera XT kit, Illumina, San Diego, CA, USA) as previously described (86). Libraries were pooled and sequenced to a depth of ∼2.5 million paired-end reads (2× 150 bp) on a NextSeq500 high-output platform (Illumina, San Diego, CA, USA). Adapters were removed from demultiplexed reads with Trimmomatic 0.38 (leading, 10; trailing, 10; sliding window, 4:15; minimum length, 60) (87). Paired ends were fixed with custom Python scripts. Trimmed reads were assembled into genomes with Unicycler 0.4.7 with default parameters (88).

FLAC genomes were sequenced and assembled as previously described (89). All WUMAC and FLAC assemblies were queried for contamination and completeness with CheckM 1.0.7 (90), and all assemblies with >95% completeness, <5% strain heterogeneity, and <2% contamination were retained.

### Retrieval of publicly available genomes.

To assemble a comprehensive MAC cohort for comparative genomic analysis, all 278 publicly available genomes for M. avium, M. intracellulare (including those formerly classified as *M. chimaera* and *M*. *yongonense* [12]), *M. colombiense*, *M. arosiense*, *M. vulneris*, *M. bouchedurhonense*, *M. timonense*, *M. marseillense*, *M. paraintracellulare*, and M. leprae*murium* were retrieved via the GenBank FTP portal. Genomes excluded from RefSeq (e.g., due to “fragmented assembly” or “many frameshifted proteins”) were discarded. All assemblies were queried for contamination and completeness with CheckM (91), and those with <95% completeness, >5% strain heterogeneity, or >2% contamination were discarded. A total of 126 high-quality genomes were retained (see Table S1 in the supplemental material). To assemble a database of mycobacterial index strains, all 96 representative and reference strains from the genera Mycobacterium and *Mycobacteroides* were similarly retrieved via the GenBank FTP portal. No genomes from this data set were discarded. Existing GenBank nomenclature for MAC species and M. avium substrains was retained.

### Species and strain profiling of the MAC cohort.

Pairwise average nucleotide identity (ANI) was calculated for all MAC isolates and between WUMAC-027/WUMAC-065 and all mycobacterial representative/reference strains with pyani 0.2.7 (MUMmer mode; ANIm) (92). MAC genome assemblies were annotated with Prokka 1.13.7 (minimum contig length, 500) (93). ANI matrices were visualized with the R package pheatmap. Using annotated proteomes (Prokka .gff files) as the input, core genome alignments and gene presence/absence matrices were generated with Roary 3.12.0 (PRANK alignment; no splitting of paralogs; core gene threshold, 99%) (94). From this core genome alignment, genome lineages were identified with fastGEAR (95), and a phylogenetic tree was built with RAxML 8.2.11 (GTRGAMMA model, 100 rapid bootstrap searches, 20 maximum likelihood searches) (96). Phylogenetic trees were visualized and annotated with metadata in iTOL v4 (89). Using the same core genome alignment as the input, a phylogenetic network was constructed with SplitsTree4 (NeighborNet mode) (18). Ordination of isolates by pangenome content was conducted with the R package Rtsne (perplexity, 30), using a Roary gene/presence absence matrix as the input. Pangenome plots were generated in R with the supplemental Roary script create_pan_genome_plots. Jaccard distance was calculated from the Roary gene presence/absence matrix using the R package vegan.

### Functional profiling of the MAC cohort.

Genes differentially abundant in human pulmonary isolates, relative to those of MAC isolates from all other sources, were identified with Scoary 1.6.16 (97). To avoid spurious false positives, counts of paralogs (e.g., XerC_1, XerC_2, XerC_3, etc.) were collapsed into single annotations (e.g., tyrosine recombinase XerC) prior to calculation of enrichment. Scoary hits were also validated via sequence similarity. A BLAST database was built from protein sequences of genes enriched in pulmonary isolates, and nonpulmonary proteomes (Prokka .faa files) were queried against this database, which yielded only expected matches. Top Scoary hits were binned into clusters of orthologous groups (COGs) (98) with eggNOG mapper v2 (91, 99) and compared to the annotated proteome of the representative M. avium strain OCU464. General or unidentified hits (COG categories R and S) were discarded. Regions of foreign DNA within MAC genomes were identified with IslandPath-DIMOB (100) within the IslandViewer4 suite (101), using each species’ representative NCBI strain (M. avium OCU464, M. intracellulare ATCC 13950, or *M. colombiense* CECT3035) as the reference for contig alignment. Predicted regions of foreign DNA were parsed to compare types of mobility regions (counts of annotated transposases, integrases, and recombinases in these regions), the total burdens of foreign DNA, and the distributions of specific genes across MAC species, as determined by Prokka (93). Virulence factors were identified by querying MAC isolates’ proteomes against a custom VF database containing all representative VF sequences from PATRIC_VF, VFDB, and Victors (45–47). Briefly, VF protein sequences were downloaded from PATRIC (45), clustered with MMseqs212–113e3 (identity, 0.9; coverage, 0.8) (102), and converted into a BLAST database. Annotated proteomes (Prokka .faa files) were queried against this database, and for each MAC protein, the top hit was retained if it exceeded 80% coverage and identity. High-quality hits with a corresponding PATRIC annotation were retained and visualized with the R package pheatmap. Antibiotic resistance genes were identified by screening MAC genomes against sequences in the MEGARes 2.0 database (58) with ABRicate (103). Published point mutations in *rrs* and *rrl* conferring resistance to aminoglycosides and macrolides, respectively (56, 57), were identified by manual inspection of these gene sequences for each isolate.

### Strain tracking of WUMAC isolates.

Phylogenies for all pulmonary M. avium isolates (*n* = 56) and all M. intracellulare isolates (*n* = 44) were generated with RAxML (96) and visualized with iTOL (89) as described above. Separate core genome alignments were created with Roary for all M. avium isolates (*n* = 25) and all M. intracellulare isolates (*n* = 16) from the WUMAC and FLAC cohorts. Pairwise SNP distance matrices were created from these WUMAC/FLAC core genome alignments with snp-dists (104) and visualized with the R package ggplot2. To construct whole-genome SNP alignments, reads from all WUMAC/FLAC M. avium and M. intracellulare isolates were aligned with the representative strains M. avium OCU464 and M. intracellulare ATCC 13950, respectively, with Snippy (105).

### Data availability.

WUMAC and FLAC genomes have been deposited in the NCBI with BioProject accession numbers PRJNA682417 and PRJNA315990, respectively.
