# Supplementary material for: Comparative Genomics of Mycobacterium avium Complex Reveals Signatures of Environment-Specific Adaptation and Community Acquisition
Source: mSystems. 2021 Oct 19;6(5):e01194-21. doi: 10.1128/mSystems.01194-21 (PMC8525567; doi:10.1128/mSystems.01194-21)
Supplement: TABLE S1 [file msystems.01194-21-st001.docx]

**Supplemental Table 1**

| **BioSample** | **Name** | **Category** | **Source** |
| --- | --- | --- | --- |
| SAMN06064229 | Mycobacterium arosiense ATCC BAA-1401 |  |  |
| SAMN02470593 | Mycobacterium avium 10-5581 | Animal | Elephant; United States |
| SAMN02603981 | Mycobacterium avium 104 | Human disseminated | Blood |
| SAMN07528777 | Mycobacterium avium FLAC0059 | Human pulmonary | Pulmonary clinical isolate; United States |
| SAMN07528781 | Mycobacterium avium FLAC0117 | Human pulmonary | Pulmonary clinical isolate; United States |
| SAMN07528782 | Mycobacterium avium FLAC0130 | Human pulmonary | Pulmonary clinical isolate; United States |
| SAMN07528784 | Mycobacterium avium FLAC0146 | Human pulmonary | Pulmonary clinical isolate; United States |
| SAMN07528785 | Mycobacterium avium FLAC0155 | Human pulmonary | Pulmonary clinical isolate; United States |
| SAMN07528786 | Mycobacterium avium FLAC0161 | Human pulmonary | Pulmonary clinical isolate; United States |
| SAMN07528788 | Mycobacterium avium FLAC0165 | Human pulmonary | Pulmonary clinical isolate; United States |
| SAMN07528791 | Mycobacterium avium FLAC0216 | Human pulmonary | Pulmonary clinical isolate; United States |
| SAMN07528792 | Mycobacterium avium FLAC0256 | Human pulmonary | Pulmonary clinical isolate; United States |
| SAMN07528793 | Mycobacterium avium FLAC0257 | Human pulmonary | Pulmonary clinical isolate; United States |
| SAMN07528794 | Mycobacterium avium FLAC0260 | Human pulmonary | Pulmonary clinical isolate; United States |
| SAMN07528795 | Mycobacterium avium FLAC0346 | Human pulmonary | Pulmonary clinical isolate; United States |
| SAMN07528796 | Mycobacterium avium FLAC0351 | Human pulmonary | Pulmonary clinical isolate; United States |
| SAMN07528797 | Mycobacterium avium FLAC0371 | Human pulmonary | Pulmonary clinical isolate; United States |
| SAMN07528798 | Mycobacterium avium FLAC0376 | Human pulmonary | Pulmonary clinical isolate; United States |
| SAMN08892076 | Mycobacterium avium HJW | Animal | Cow stool; China |
| SAMN04325949 | Mycobacterium avium RCAD0278 | Animal | Duck; China |
| SAMN02470595 | Mycobacterium avium subsp. avium 10-9275 | Animal | Red-tailed hawk; United States |
| SAMN02470543 | Mycobacterium avium subsp. avium ATCC 25291 | Animal | Hen |
| SAMN13352280 | Mycobacterium avium subsp. avium DSM 44156 | Animal | Junglefowl liver |
| SAMN02756839 | Mycobacterium avium subsp. hominissuis 101 | Human disseminated | Blood; United States |
| SAMN11403486 | Mycobacterium avium subsp. hominissuis 101034 | Human pulmonary | Pulmonary clinical isolate; United States |
| SAMN11403599 | Mycobacterium avium subsp. hominissuis 101115 | Human pulmonary | Pulmonary clinical isolate; United States |
| SAMN11403589 | Mycobacterium avium subsp. hominissuis 101174 | Human pulmonary | Pulmonary clinical isolate; United States |
| SAMN07184188 | Mycobacterium avium subsp. hominissuis 11 | Human disseminated | Bone marrow; Taiwan |
| SAMEA3925670 | Mycobacterium avium subsp. hominissuis 12 062 | Human disseminated | Belgium |
| SAMN02756836 | Mycobacterium avium subsp. hominissuis A5 | Human disseminated | Blood; United States |
| SAMN05412758 | Mycobacterium avium subsp. hominissuis CAM177 | Human pulmonary | Pulmonary clinical isolate; Japan |
| SAMN05412756 | Mycobacterium avium subsp. hominissuis CAM57 | Human pulmonary | Pulmonary clinical isolate; Japan |
| SAMD00076682 | Mycobacterium avium subsp. hominissuis DH-5 | Human pulmonary | Pulmonary clinical isolate; Japan |
| SAMD00076683 | Mycobacterium avium subsp. hominissuis DH-8-4 | Human pulmonary | Pulmonary clinical isolate; Japan |
| SAMN04566723 | Mycobacterium avium subsp. hominissuis E-128 | Environmental | Soil; Germany |
| SAMD00076684 | Mycobacterium avium subsp. hominissuis HF-4 | Human pulmonary | Pulmonary clinical isolate; Japan |
| SAMN05506874 | Mycobacterium avium subsp. hominissuis HP17 | Human pulmonary | Pulmonary clinical isolate; Japan |
| SAMD00076656 | Mycobacterium avium subsp. hominissuis IH-065 | Human pulmonary | Pulmonary clinical isolate; Japan |
| SAMD00076657 | Mycobacterium avium subsp. hominissuis IH-068 | Human pulmonary | Pulmonary clinical isolate; Japan |
| SAMD00076660 | Mycobacterium avium subsp. hominissuis IH-217 | Human pulmonary | Pulmonary clinical isolate; Japan |
| SAMD00076663 | Mycobacterium avium subsp. hominissuis IH-532 | Human pulmonary | Pulmonary clinical isolate; Japan |
| SAMD00076662 | Mycobacterium avium subsp. hominissuis IH-550 | Human pulmonary | Pulmonary clinical isolate; Japan |
| SAMD00076664 | Mycobacterium avium subsp. hominissuis IH-560 | Human pulmonary | Pulmonary clinical isolate; Japan |
| SAMD00184123 | Mycobacterium avium subsp. hominissuis JP-H-1 | Animal | Horse disseminated |
| SAMD00076671 | Mycobacterium avium subsp. hominissuis Kin-16 | Human pulmonary | Pulmonary clinical isolate; Japan |
| SAMEA3925668 | Mycobacterium avium subsp. hominissuis LYM122 | Animal | Pig; Belgium |
| SAMN09074392 | Mycobacterium avium subsp. hominissuis MAC109 | Human disseminated | Blood |
| SAMN04229409 | Mycobacterium avium subsp. hominissuis MAH-E-101-6 | Environmental | Dust; Germany |
| SAMN04229410 | Mycobacterium avium subsp. hominissuis MAH-E-104-1 | Environmental | Soil; Germany |
| SAMN04229411 | Mycobacterium avium subsp. hominissuis MAH-E-106 | Environmental | Soil; Germany |
| SAMN04229412 | Mycobacterium avium subsp. hominissuis MAH-E-108 | Environmental | Dust; Germany |
| SAMN07808707 | Mycobacterium avium subsp. hominissuis MAH-E-14-1 | Environmental | Soil; Germany |
| SAMN04229415 | Mycobacterium avium subsp. hominissuis MAH-E-149-2 | Environmental | Soil; Germany |
| SAMN04229404 | Mycobacterium avium subsp. hominissuis MAH-E-61-1t | Environmental | Dust; Germany |
| SAMN04229405 | Mycobacterium avium subsp. hominissuis MAH-E-61-1y | Environmental | Dust; Germany |
| SAMN04229407 | Mycobacterium avium subsp. hominissuis MAH-E-63-1 | Environmental | Dust; Germany |
| SAMN04229397 | Mycobacterium avium subsp. hominissuis MAH-E-82-7 | Environmental | Dust; Germany |
| SAMN04229399 | Mycobacterium avium subsp. hominissuis MAH-E-83-1 | Environmental | Dust; Germany |
| SAMN04229400 | Mycobacterium avium subsp. hominissuis MAH-E-88-1 | Environmental | Dust; Germany |
| SAMN10955938 | Mycobacterium avium subsp. hominissuis mc2 2500 | Human pulmonary | Pulmonary clinical isolate; United States |
| SAMD00076676 | Mycobacterium avium subsp. hominissuis NN-108 | Human pulmonary | Pulmonary clinical isolate; Japan |
| SAMN03013888 | Mycobacterium avium subsp. hominissuis OCU464 | Human pulmonary | Pulmonary clinical isolate; Japan |
| SAMN05821954 | Mycobacterium avium subsp. hominissuis OCU556 | Human disseminated | Lymph node; Japan |
| SAMN05862070 | Mycobacterium avium subsp. hominissuis OCU873s P7 4s | Human pulmonary | Pulmonary clinical isolate; Japan |
| SAMN05862128 | Mycobacterium avium subsp. hominissuis OCU901s S2 2s | Human pulmonary | Pulmonary clinical isolate; Japan |
| SAMD00061038 | Mycobacterium avium subsp. hominissuis TH135 | Human pulmonary | Pulmonary clinical isolate; Japan |
| SAMD00076687 | Mycobacterium avium subsp. hominissuis Tone-1 | Human pulmonary | Pulmonary clinical isolate; Japan |
| SAMD00076689 | Mycobacterium avium subsp. hominissuis Tone-12 | Human pulmonary | Pulmonary clinical isolate; Japan |
| SAMD00076690 | Mycobacterium avium subsp. hominissuis Tone-13 | Human pulmonary | Pulmonary clinical isolate; Japan |
| SAMD00076691 | Mycobacterium avium subsp. hominissuis Tone-16 | Human pulmonary | Pulmonary clinical isolate; Japan |
| SAMD00076688 | Mycobacterium avium subsp. hominissuis Tone-5 | Human pulmonary | Pulmonary clinical isolate; Japan |
| SAMD00076693 | Mycobacterium avium subsp. hominissuis TR-M-2 | Human pulmonary | Pulmonary clinical isolate; Japan |
| SAMD00076695 | Mycobacterium avium subsp. hominissuis TR-M-4 | Human pulmonary | Pulmonary clinical isolate; Japan |
| SAMD00076696 | Mycobacterium avium subsp. hominissuis TR-M-5 | Human pulmonary | Pulmonary clinical isolate; Japan |
| SAMN06272994 | Mycobacterium avium subsp. paratuberculosis A3 | Animal | Cow stool; Portugal |
| SAMN14764132 | Mycobacterium avium subsp. paratuberculosis DSM 44135 | Animal | Cow; Germany |
| SAMN03252100 | Mycobacterium avium subsp. paratuberculosis E1 | Animal | Animal stool; Egypt |
| SAMN03252736 | Mycobacterium avium subsp. paratuberculosis E93 | Animal | Animal stool; Egypt |
| SAMN06173318 | Mycobacterium avium subsp. paratuberculosis FDAARGOS 305 | Animal | Dairy animal; United States |
| SAMN07249768 | Mycobacterium avium subsp. paratuberculosis JII-1961 | Animal | Cow lymph node; Germany |
| SAMN07204620 | Mycobacterium avium subsp. paratuberculosis JIII-386 | Animal | Sheep ileum; Germany |
| SAMN02604086 | Mycobacterium avium subsp. paratuberculosis K-10 | Animal | Dairy animal |
| SAMN04544372 | Mycobacterium avium subsp. paratuberculosis MAP | Animal | Cow; India |
| SAMN02603716 | Mycobacterium avium subsp. paratuberculosis MAP4 | Human disseminated | Breast milk |
| SAMN10410800 | Mycobacterium avium subsp. paratuberculosis MAPK CN4 13 | Animal | Cow; South Korea |
| SAMN10337859 | Mycobacterium avium subsp. paratuberculosis MAPK CN7 15 | Animal | Cow; South Korea |
| SAMN10337856 | Mycobacterium avium subsp. paratuberculosis MAPK CN9 15 | Animal | Cow stool; South Korea |
| SAMN10410803 | Mycobacterium avium subsp. paratuberculosis MAPK JB16 15 | Animal | Cow; South Korea |
| SAMN10410790 | Mycobacterium avium subsp. paratuberculosis MAPK JJ1 13 | Animal | Cow; South Korea |
| SAMN03892587 | Mycobacterium avium subsp. paratuberculosis NL 89C | Animal | Dairy animal; Canada |
| SAMN03892589 | Mycobacterium avium subsp. paratuberculosis NL 93B | Animal | Dairy animal; Canada |
| SAMN03892638 | Mycobacterium avium subsp. paratuberculosis NL 95A | Animal | Dairy animal; Canada |
| SAMN03892639 | Mycobacterium avium subsp. paratuberculosis NL 95B | Animal | Dairy animal; Canada |
| SAMN03892640 | Mycobacterium avium subsp. paratuberculosis NL 95E | Animal | Dairy animal; Canada |
| SAMN03892643 | Mycobacterium avium subsp. paratuberculosis NL 96E | Animal | Dairy animal; Canada |
| SAMN02470597 | Mycobacterium avium subsp. paratuberculosis S397 | Animal | Sheep; United States |
| SAMN02469426 | Mycobacterium avium subsp. paratuberculosis S5 | Animal | Goat; India |
| SAMN10396996 | Mycobacterium avium subsp. paratuberculosis Telford | Animal | Sheep stool; Australia |
| SAMN16988975 | Mycobacterium avium WUMAC-013 | Human pulmonary | Pulmonary clinical isolate; United States |
| SAMN16988976 | Mycobacterium avium WUMAC-014 | Human pulmonary | Pulmonary clinical isolate; United States |
| SAMN16988979 | Mycobacterium avium WUMAC-019 | Human pulmonary | Pulmonary clinical isolate; United States |
| SAMN16988980 | Mycobacterium avium WUMAC-020 | Human pulmonary | Pulmonary clinical isolate; United States |
| SAMN16988981 | Mycobacterium avium WUMAC-021 | Human pulmonary | Pulmonary clinical isolate; United States |
| SAMN16988982 | Mycobacterium avium WUMAC-022 | Human pulmonary | Pulmonary clinical isolate; United States |
| SAMN16988986 | Mycobacterium avium WUMAC-026 | Human pulmonary | Pulmonary clinical isolate; United States |
| SAMN16988993 | Mycobacterium avium WUMAC-035 | Human pulmonary | Pulmonary clinical isolate; United States |
| SAMN16988997 | Mycobacterium avium WUMAC-060 | Human pulmonary | Pulmonary clinical isolate; United States |
| SAMN16988998 | Mycobacterium avium WUMAC-062 | Human pulmonary | Pulmonary clinical isolate; United States |
| SAMN16988999 | Mycobacterium avium WUMAC-064 | Human pulmonary | Pulmonary clinical isolate; United States |
| SAMN02419652 | Mycobacterium avium XTB13-223 | Human disseminated | Stool; Belarus |
| SAMN04691980 | Mycobacterium colombiense 852002-51834 SCH5396731 |  |  |
| SAMN00622208 | Mycobacterium colombiense CECT 3035 |  |  |
| SAMN06760893 | Mycobacterium colombiense CECT 3035 CECT 3035 |  |  |
| SAMN09476219 | Mycobacterium colombiense GF28 |  |  |
| SAMN09476220 | Mycobacterium colombiense GF76 |  |  |
| SAMN04634276 | Mycobacterium colombiense IS-2214 |  |  |
| SAMN04634270 | Mycobacterium colombiense NS-7390 |  |  |
| SAMN02470538 | Mycobacterium intracellulare ATCC 13950 McGill |  |  |
| SAMN02603186 | Mycobacterium intracellulare ATCC 13950 Seoul |  |  |
| SAMEA5546145 | Mycobacterium intracellulare CSURP8077 |  |  |
| SAMN04634222 | Mycobacterium intracellulare E2190 |  |  |
| SAMN07528783 | Mycobacterium intracellulare FLAC0133 |  |  |
| SAMN07528789 | Mycobacterium intracellulare FLAC0181 |  |  |
| SAMN07528790 | Mycobacterium intracellulare FLAC0204 |  |  |
| SAMD00036621 | Mycobacterium intracellulare M.i.198 |  |  |
| SAMN02603184 | Mycobacterium intracellulare MOTT-02 |  |  |
| SAMN13191638 | Mycobacterium intracellulare subsp. chimaera AUSMDU00007395 |  |  |
| SAMN05824346 | Mycobacterium intracellulare subsp. chimaera CDC 2015-22-71 |  |  |
| SAMEA5546144 | Mycobacterium intracellulare subsp. chimaera CSURP8078 |  |  |
| SAMN01174703 | Mycobacterium intracellulare subsp. chimaera DSM 44623 Leibniz |  |  |
| SAMN04216918 | Mycobacterium intracellulare subsp. chimaera DSM 44623 Trento |  |  |
| SAMN07528778 | Mycobacterium intracellulare subsp. chimaera FLAC0063 |  |  |
| SAMN07528779 | Mycobacterium intracellulare subsp. chimaera FLAC0067 |  |  |
| SAMN07528780 | Mycobacterium intracellulare subsp. chimaera FLAC0070 |  |  |
| SAMN05918811 | Mycobacterium intracellulare subsp. chimaera JCM 14737 |  |  |
| SAMEA4521417 | Mycobacterium intracellulare subsp. chimaera MC045 |  |  |
| SAMN04032784 | Mycobacterium intracellulare subsp. chimaera MCIMRL2 |  |  |
| SAMN04032785 | Mycobacterium intracellulare subsp. chimaera MCIMRL4 |  |  |
| SAMN04032786 | Mycobacterium intracellulare subsp. chimaera MCIMRL6 |  |  |
| SAMN07274465 | Mycobacterium intracellulare subsp. chimaera SJ42 |  |  |
| SAMN07606280 | Mycobacterium intracellulare subsp. chimaera WCHMC000001 |  |  |
| SAMN07606281 | Mycobacterium intracellulare subsp. chimaera WCHMC000030 |  |  |
| SAMN07606282 | Mycobacterium intracellulare subsp. chimaera WCHMC000032 |  |  |
| SAMN16988995 | Mycobacterium intracellulare subsp. chimaera WUMAC-057 |  |  |
| SAMN16988996 | Mycobacterium intracellulare subsp. chimaera WUMAC-058 |  |  |
| SAMN04525300 | Mycobacterium intracellulare subsp. chimaera ZUERICH-1 |  |  |
| SAMN04526974 | Mycobacterium intracellulare subsp. chimaera ZUERICH-2 |  |  |
| SAMN02603610 | Mycobacterium intracellulare subsp. intracellulare MTCC 9506 |  |  |
| SAMN02603187 | Mycobacterium intracellulare subsp. yongonense 05-1390 |  |  |
| SAMN04390159 | Mycobacterium intracellulare subsp. yongonense Asan 36527 |  |  |
| SAMN16988977 | Mycobacterium intracellulare WUMAC-015 |  |  |
| SAMN16988978 | Mycobacterium intracellulare WUMAC-016 |  |  |
| SAMN16988983 | Mycobacterium intracellulare WUMAC-023 |  |  |
| SAMN16988984 | Mycobacterium intracellulare WUMAC-024 |  |  |
| SAMN16988987 | Mycobacterium intracellulare WUMAC-027 |  |  |
| SAMN16988988 | Mycobacterium intracellulare WUMAC-028 |  |  |
| SAMN16988989 | Mycobacterium intracellulare WUMAC-029 |  |  |
| SAMN16988990 | Mycobacterium intracellulare WUMAC-031 |  |  |
| SAMN16988991 | Mycobacterium intracellulare WUMAC-032 |  |  |
| SAMN16988992 | Mycobacterium intracellulare WUMAC-033 |  |  |
| SAMN16988994 | Mycobacterium intracellulare WUMAC-056 |  |  |
| SAMN06841017 | Mycobacterium lepraemurium Hawaii |  |  |
| SAMN06064246 | Mycobacterium marseillense DSM 45437 |  |  |
| SAMN07528776 | Mycobacterium marseillense FLAC0026 |  |  |
| SAMN16989000 | Mycobacterium marseillense WUMAC-066 |  |  |
| SAMN16988985 | Mycobacterium sp. WUMAC-025 |  |  |
| SAMN16989001 | Mycobacterium sp. WUMAC-067 |  |  |
| SAMN04634267 | Mycobacterium vulneris ACS4093 |  |  |
| SAMN04634259 | Mycobacterium vulneris ACS5020 |  |  |
| SAMN04634260 | Mycobacterium vulneris ICS2043 |  |  |
