# Supplementary material for: Comparative Genomics of Mycobacterium avium Complex Reveals Signatures of Environment-Specific Adaptation and Community Acquisition
Source: mSystems. 2021 Oct 19;6(5):e01194-21. doi: 10.1128/mSystems.01194-21 (PMC8525567; doi:10.1128/mSystems.01194-21)
Supplement: TABLE S2 [file msystems.01194-21-st002.docx]

**Supplemental Table 2**

| **Isolate** | **MALDI-TOF identification** | **ANI identification** | **Patient** | **Year collected** | **Age** | **Gender** | **Predisposing factors &**  **co-morbidities** |
| --- | --- | --- | --- | --- | --- | --- | --- |
| WUMAC-013 | *M. avium* | *M. avium* | WUMAC MA patient 1 | 2016 | 50-60 | Male | End-stage sarcoidosis, PHTN, GERD |
| WUMAC-014 | *M. avium* | *M. avium* | WUMAC MA patient 1 | 2018 | 50-60 | Male | End-stage sarcoidosis, PHTN, GERD |
| WUMAC-015 | *M. chimaera-intracellulare* | *M. intracellulare* | WUMAC MI patient 1 | 2016 | 10-20 | Female | CF, pancreatic insufficiency, ABPA |
| WUMAC-016 | *M. chimaera-intracellulare* | *M. intracellulare* | WUMAC MI patient 1 | 2018 | 10-20 | Female | CF, pancreatic insufficiency, ABPA |
| WUMAC-019 | *M. avium* | *M. avium* | WUMAC MA patient 2 | 2016 | 40-50 | Female | CF |
| WUMAC-020 | *M. avium* | *M. avium* | WUMAC MA patient 2 | 2018 | 40-50 | Female | CF |
| WUMAC-021 | *M. avium* | *M. avium* | WUMAC MA patient 3 | 2016 | 30-40 | Female | COPD, PLWHIV, substance abuse |
| WUMAC-022 | *M. avium* | *M. avium* | WUMAC MA patient 3 | 2017 | 30-40 | Female | COPD, PLWHIV, substance abuse |
| WUMAC-023 | *M. chimaera-intracellulare* | *M. intracellulare* | WUMAC MI patient 3 | 2018 | 60-70 | Female | Sarcoidosis |
| WUMAC-024 | *M. chimaera-intracellulare* | *M. intracellulare* | WUMAC MI patient 3 | 2019 | 60-70 | Female | Sarcoidosis |
| WUMAC-025 | *M. avium-intracellulare* complex | Novel species | WUMAC MN patient 1 | 2018 | 50-60 | Male | GERD, sarcoidosis, PHTN, lung transplant |
| WUMAC-026 | *M. avium-intracellulare* complex | *M. avium* | WUMAC MA patient 4 | 2013 | 50-60 | Female | Bronchiectasis |
| WUMAC-027 | *M. chimaera-intracellulare* | *M. intracellulare* | WUMAC MI patient 4 | 2018 | 80-90 | Female | GERD, IBD, PHTN, bronchiectasis, COPD |
| WUMAC-028 | *M. chimaera-intracellulare* | *M. intracellulare* | WUMAC MI patient 4 | 2017 | 80-90 | Female | GERD, IBD, PHTN, bronchiectasis, COPD |
| WUMAC-029 | *M. avium-intracellulare* complex | *M. intracellulare* | WUMAC MI patient 8 | 2015 | 70-80 | Female | CHF, COPD |
| WUMAC-031 | *M. chimaera-intracellulare* | *M. intracellulare* | WUMAC MI patient 2 | 2019 | 60-70 | Female | Bronchiectasis, metastatic cancer |
| WUMAC-032 | *M. chimaera-intracellulare* | *M. intracellulare* | WUMAC MI patient 2 | 2019 | 60-70 | Female | Bronchiectasis, metastatic cancer |
| WUMAC-033 | *M. avium-intracellulare* complex | *M. intracellulare* | WUMAC MI patient 7 | 2015 | 30-40 | Male | CF, CKD, DM, lung transplant |
| WUMAC-035 | *M. avium-intracellulare* complex | *M. avium* | WUMAC MA patient 5 | 2009 | 70-80 | Female | Bronchiectasis, RA |
| WUMAC-056 | *M. avium-intracellulare* complex | *M. intracellulare* | WUMAC MI patient 6 | 2017 | 60-70 | Female | Bronchiectasis |
| WUMAC-057 | *M. chimaera-intracellulare* | *M. intracellulare* | WUMAC MI patient 5 | 2018 | 60-70 | Female | Bronchiectasis, breast cancer in remission |
| WUMAC-058 | *M. chimaera-intracellulare* | *M. intracellulare* | WUMAC MI patient 5 | 2017 | 60-70 | Female | Bronchiectasis, breast cancer in remission |
| WUMAC-060 | *M. avium-intracellulare* complex | *M. avium* | WUMAC MA patient 6 | 2014 | 70-80 | Female | GERD, RA |
| WUMAC-062 | *M. avium-intracellulare* complex | *M. avium* | WUMAC MA patient 7 | 2016 | 10-20 | Male | CF |
| WUMAC-064 | *M. avium-intracellulare* complex | *M. avium* | WUMAC MA patient 8 | 2016 | 40-50 | Female | DM, PHTN |
| WUMAC-066 | *M. avium-intracellulare* complex | *M. marseillense* | WUMAC MM patient 1 | 2016 | 80-90 | Male | Bronchiectasis, COPD |
| WUMAC-067 | *M. avium-intracellulare* complex | Novel species | WUMAC MN patient 1 | 2016 | 50-60 | Male | GERD, sarcoidosis, PHTN, lung transplant |
| FLAC0117 | ND | *M. avium* | FLAC MA patient 3 | 2014 | 50-60 | Female | CF, GERD |
| FLAC0130 | ND | *M. avium* | FLAC MA patient 1 | 2014 | 10-20 | Female | CF, ABPA |
| FLAC0146 | ND | *M. avium* | FLAC MA patient 4 | 2014 | 50-60 | Male | CF, GERD, CFRD |
| FLAC0155 | ND | *M. avium* | FLAC MA patient 2 | 2014 | 30-40 | Female | CF, CFRD |
| FLAC0161 | ND | *M. avium* | FLAC MA patient 5 | 2013 | 40-50 | Male | CF |
| FLAC0165 | ND | *M. avium* | FLAC MA patient 6 | 2014 | 10-20 | Female | CF, GERD |
| FLAC0216 | ND | *M. avium* | FLAC MA patient 1 | 2014 | 10-20 | Female | CF, ABPA |
| FLAC0256 | ND | *M. avium* | FLAC MA patient 7 | 2015 | 20-30 | Female | CF |
| FLAC0257 | ND | *M. avium* | FLAC MA patient 8 | 2015 | 30-40 | Female | CF |
| FLAC0260 | ND | *M. avium* | FLAC MA patient 2 | 2014 | 30-40 | Female | CF, CFRD |
| FLAC0346 | ND | *M. avium* | FLAC MA patient 9 | 2015 | 20-30 | Female | CF |
| FLAC0351 | ND | *M. avium* | FLAC MA patient 10 | 2016 | 50-60 | Male | CF, COPD, GERD, CFRD |
| FLAC0371 | ND | *M. avium* | FLAC MA patient 11 | 2016 | 50-60 | Female | CF, COPD, GERD |
| FLAC0376 | ND | *M. avium* | FLAC MA patient 12 | 2015 | 20-30 | Female | CF, GERD, CFRD |
| FLAC0133 | ND | *M. intracellulare* | FLAC MI patient 2 | 2014 | 0-10 | Male | CF, GERD |
| FLAC0181 | ND | *M. intracellulare* | FLAC MI patient 1 | 2014 | 20-30 | Male | CF, bronchiectasis, GERD |
| FLAC0204 | ND | *M. intracellulare* | FLAC MI patient 1 | 2014 | 20-30 | Male | CF, bronchiectasis, GERD |
