# Supplementary figures and images for: Comparative Genomics of Mycobacterium avium Complex Reveals Signatures of Environment-Specific Adaptation and Community Acquisition
Source: mSystems. 2021 Oct 19;6(5):e01194-21. doi: 10.1128/mSystems.01194-21 (PMC8525567; doi:10.1128/mSystems.01194-21)

# Supplemental Figure 1

A

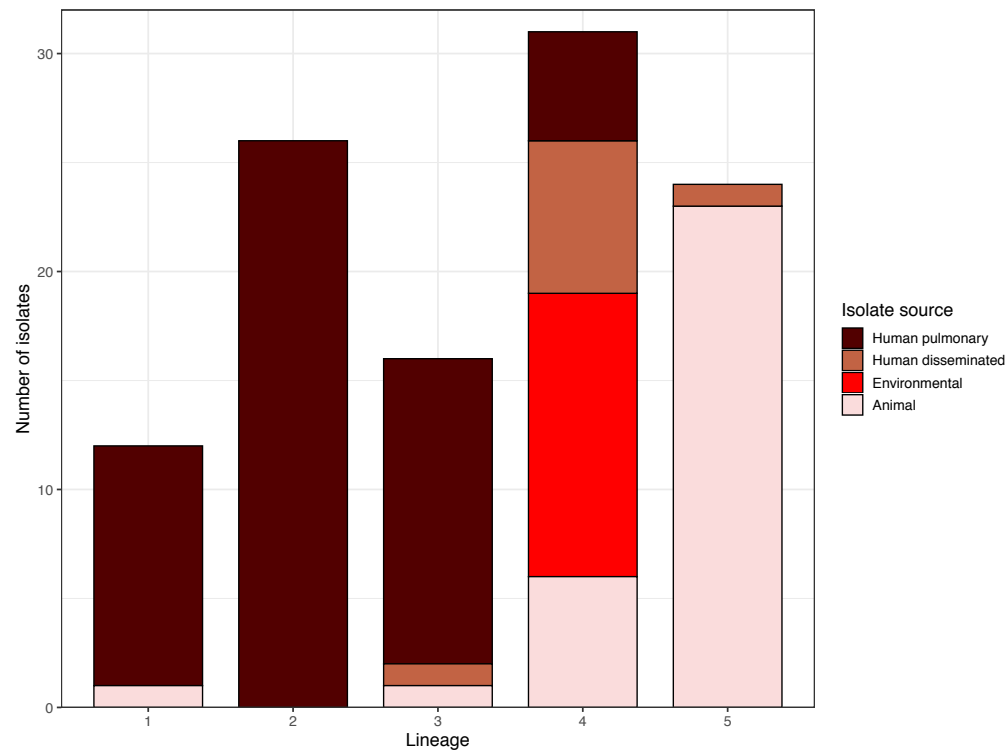

B

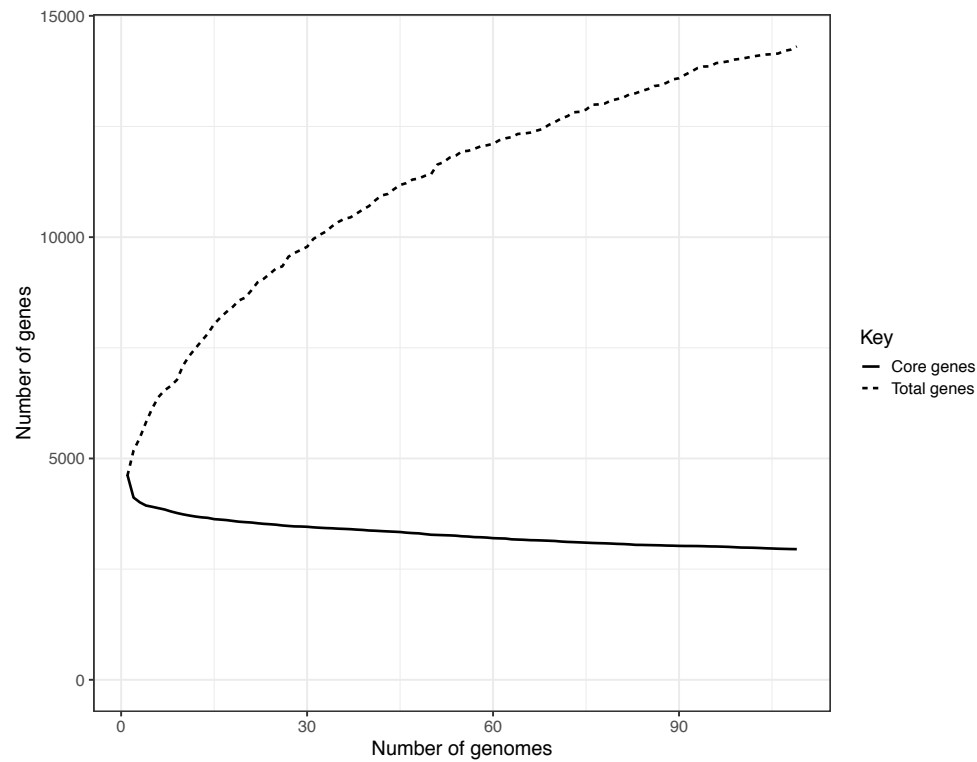

Supplement: FIG S1 [file msystems.01194-21-sf001.pdf]

Supplemental Figure 2

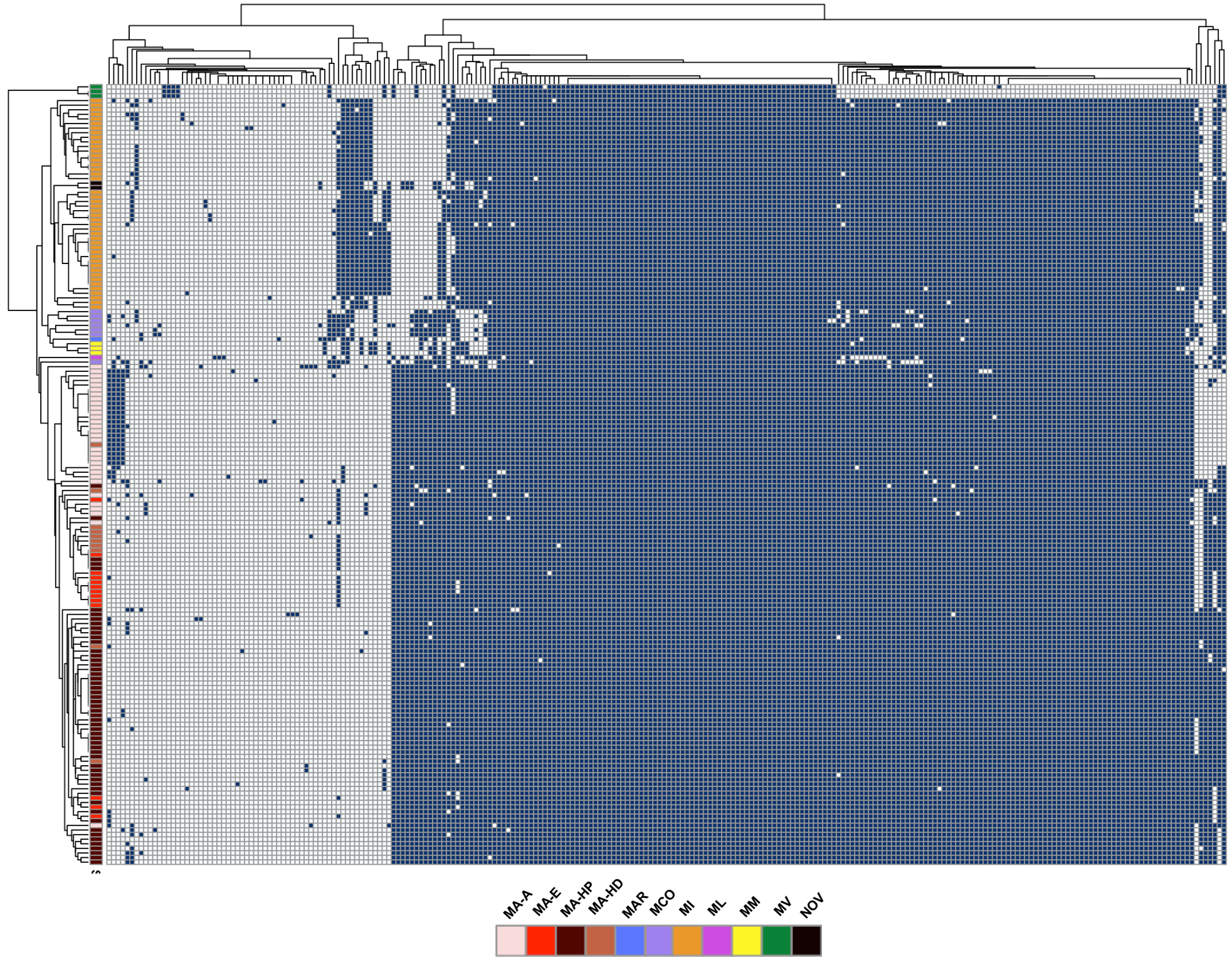

Supplement: FIG S2 [file msystems.01194-21-sf002.pdf]

Supplemental Figure 3

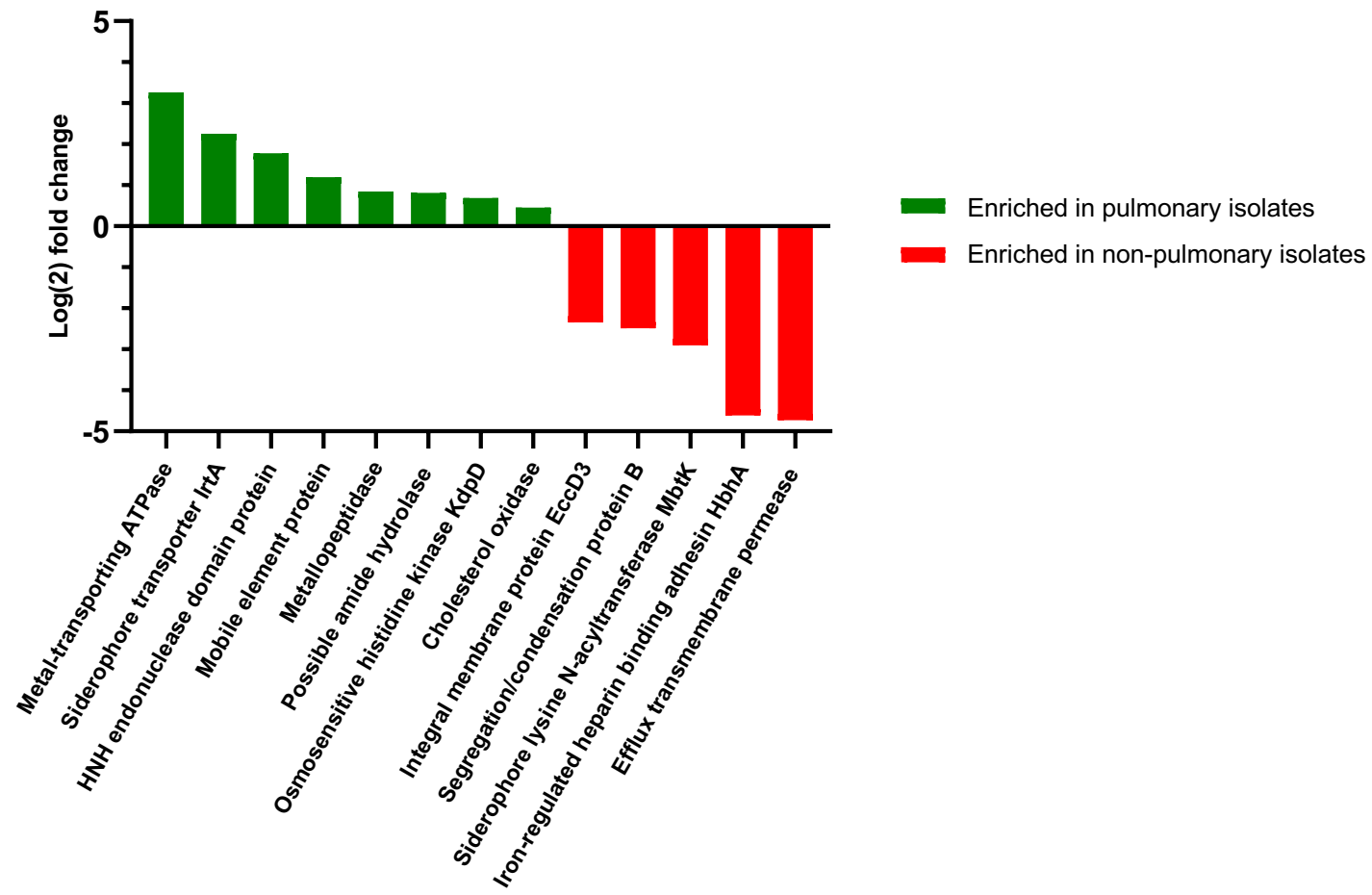

Supplement: FIG S3 [file msystems.01194-21-sf003.pdf]

Supplemental Figure 4

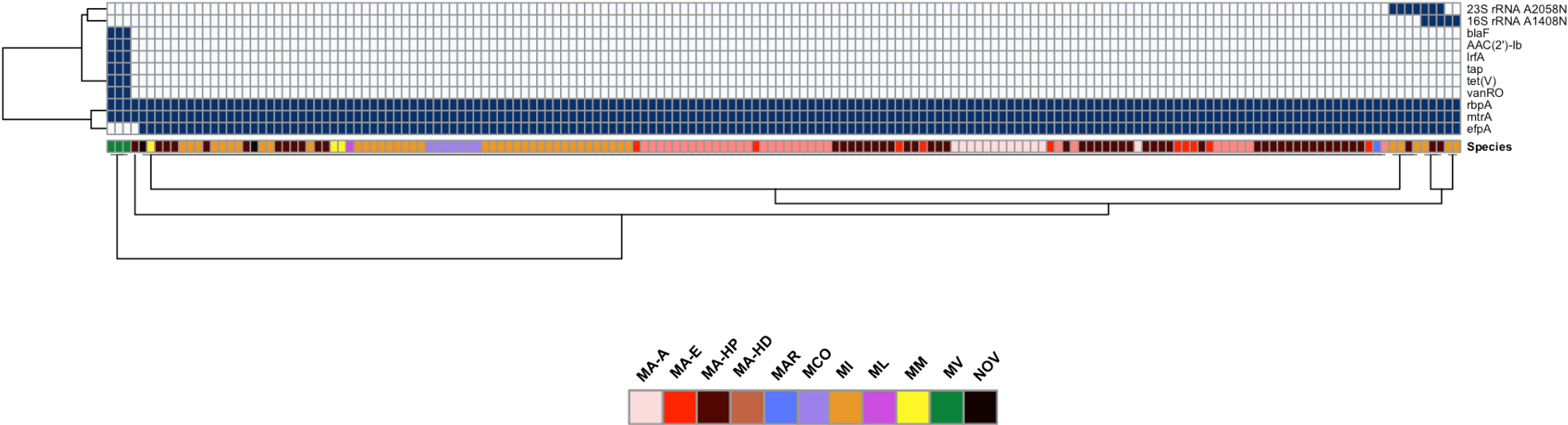

Supplement: FIG S4 [file msystems.01194-21-sf004.pdf]

Supplemental Figure 5

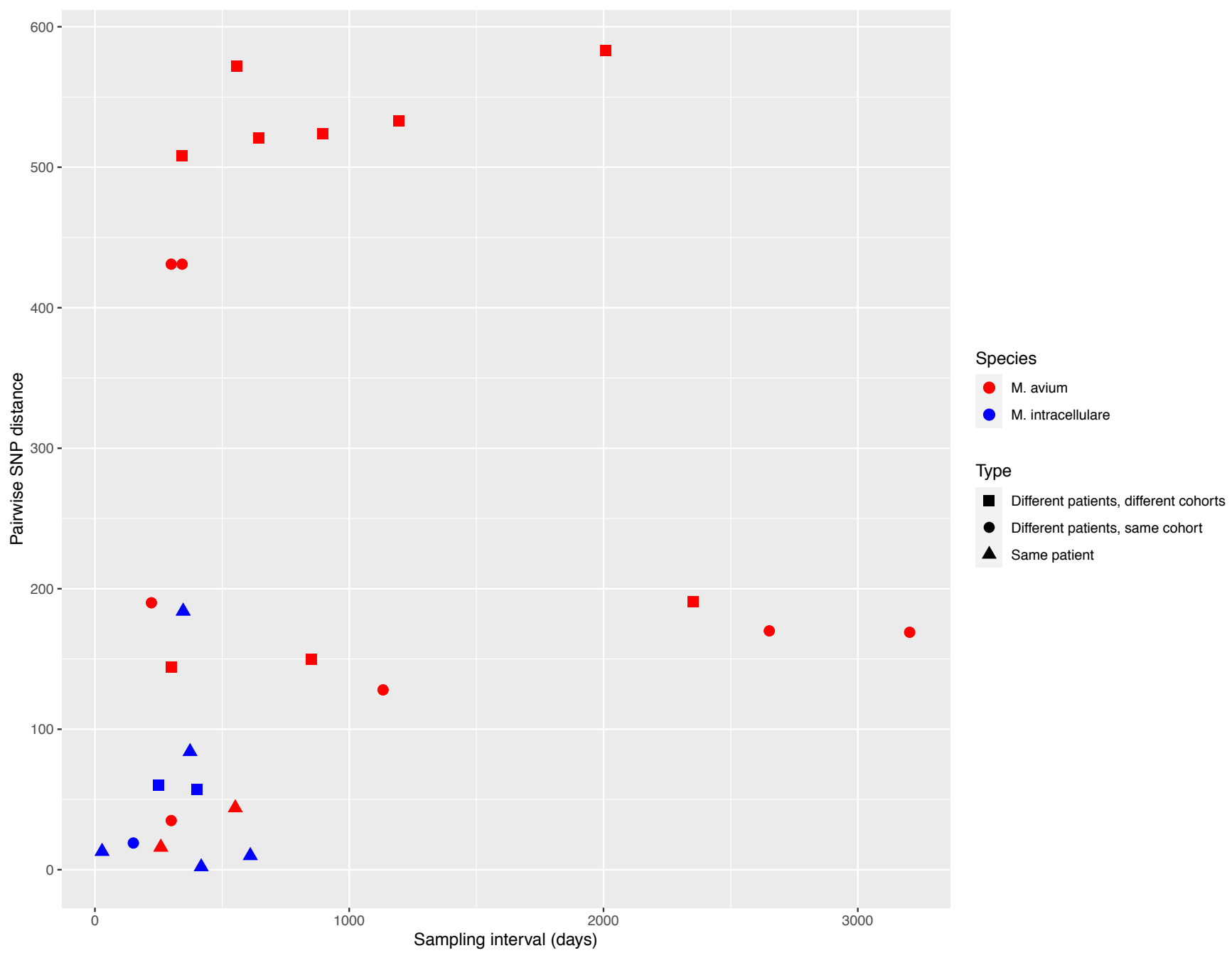

Supplement: FIG S5 [file msystems.01194-21-sf005.pdf]
