# Supplementary material for: Comparative Genomics of Mycobacterium avium Complex Reveals Signatures of Environment-Specific Adaptation and Community Acquisition
Source: mSystems. 2021 Oct 19;6(5):e01194-21. doi: 10.1128/mSystems.01194-21 (PMC8525567; doi:10.1128/mSystems.01194-21)
Supplement: TABLE S3 [file msystems.01194-21-st003.docx]

**Supplemental Table 3**

| **Gene** | **Log(2) fold change** | **P-value** |
| --- | --- | --- |
| Acyl-CoA dehydrogenase FadE34 | 5.35 | 1.36E-05 |
| IS3 family transposase IS999 | 5.35 | 1.36E-05 |
| ATP-dependent zinc metalloprotease FtsH | 4.94 | 3.61E-04 |
| Putative N-acetyl-LL-diaminopimelate aminotransferase | 4.94 | 3.61E-04 |
| IS481 family transposase ISMav3 | 4.94 | 1.40E-03 |
| (-)-trans-carveol dehydrogenase | 4.35 | 6.49E-03 |
| 1,2-epoxyphenylacetyl-CoA isomerase | 4.35 | 6.49E-03 |
| 1,4-dihydroxy-2-naphthoyl-CoA synthase | 4.35 | 6.49E-03 |
| 1,5-anhydro-D-fructose reductase | 4.35 | 6.49E-03 |
| 1,8-cineole 2-endo-monooxygenase | 4.35 | 6.49E-03 |
| 2-dehydro-3-deoxy-D-gluconate 5-dehydrogenase | 4.35 | 6.49E-03 |
| 2-hydroxy-3-oxopropionate reductase | 4.35 | 6.49E-03 |
| 2-oxo-hept-4-ene-1,7-dioate hydratase | 4.35 | 6.49E-03 |
| 2-succinyl-6-hydroxy-2,4-cyclohexadiene-1-carboxylate synthase | 4.35 | 6.49E-03 |
| 2-succinylbenzoate--CoA ligase | 4.35 | 6.49E-03 |
| 2,3-dihydroxyphenylpropionate/2,3-dihydroxicinnamic acid 1,2-dioxygenase | 4.35 | 6.49E-03 |
| 2,4-diaminopentanoate dehydrogenase | 4.35 | 6.49E-03 |
| 2,5-dichloro-2,5-cyclohexadiene-1,4-diol dehydrogenase | 4.35 | 6.49E-03 |
| 3-[(3aS,4S,7aS)-7a-methyl-1,5-dioxo-octahydro-1H-inden-4-yl]propanoyl:CoA ligase | 4.35 | 6.49E-03 |
| 3-ketoacyl-CoA thiolase | 4.35 | 6.49E-03 |
| 3-ketosteroid-9-alpha-monooxygenase, oxygenase component | 4.35 | 6.49E-03 |
| 3-oxoacyl-[acyl-carrier-protein] reductase FabG | 4.35 | 6.49E-03 |
| 3-phenylpropionate/cinnamic acid dioxygenase subunit beta | 4.35 | 6.49E-03 |
| 3-succinoylsemialdehyde-pyridine dehydrogenase | 4.35 | 6.49E-03 |
| 4-hydroxy-2-oxo-heptane-1,7-dioate aldolase | 4.35 | 6.49E-03 |
| 4-hydroxy-2-oxovalerate aldolase | 4.35 | 6.49E-03 |
| 4,5:9,10-diseco-3-hydroxy-5,9,17-trioxoandrosta-1(10),2-diene-4-oate hydrolase | 4.35 | 6.49E-03 |
| Acetyl-CoA:oxalate CoA-transferase | 4.35 | 6.49E-03 |
| Acyl-CoA dehydrogenase | 4.35 | 6.49E-03 |
| Acyl-CoA dehydrogenase FadE27 | 4.35 | 6.49E-03 |
| Acyltrehalose exporter MmpL10 | 4.35 | 6.49E-03 |
| Alcohol dehydrogenase B | 4.35 | 6.49E-03 |
| Alkanesulfonate monooxygenase | 4.35 | 6.49E-03 |
| Antitoxin RelF | 4.35 | 6.49E-03 |
| ATP-dependent RecD-like DNA helicase | 4.35 | 6.49E-03 |
| Beta-ketoadipyl-CoA thiolase | 4.35 | 6.49E-03 |
| Betaine aldehyde dehydrogenase | 4.35 | 6.49E-03 |
| Carnitinyl-CoA dehydratase | 4.35 | 6.49E-03 |
| Cinnamoyl-CoA:phenyllactate CoA-transferase | 4.35 | 6.49E-03 |
| CoA-transferase/lyase DddD | 4.35 | 6.49E-03 |
| Crotonobetainyl-CoA dehydrogenase | 4.35 | 6.49E-03 |
| D-aminopeptidase | 4.35 | 6.49E-03 |
| Deazaflavin-dependent nitroreductase | 4.35 | 6.49E-03 |
| Diacetyl reductase [(S)-acetoin forming] | 4.35 | 6.49E-03 |
| dTDP-glucose 4,6-dehydratase | 4.35 | 6.49E-03 |
| Enoyl-CoA-hydratase | 4.35 | 6.49E-03 |
| F420-dependent glucose-6-phosphate dehydrogenase | 4.35 | 6.49E-03 |
| Ferredoxin | 4.35 | 6.49E-03 |
| FMN reductase [NAD(P)H] | 4.35 | 6.49E-03 |
| Geranial dehydrogenase | 4.35 | 6.49E-03 |
| Glutaredoxin-like protein NrdH | 4.35 | 6.49E-03 |
| Haloalkane dehalogenase 2 | 4.35 | 6.49E-03 |
| HTH-type transcriptional regulator BetI | 4.35 | 6.49E-03 |
| HTH-type transcriptional repressor KstR | 4.35 | 6.49E-03 |
| HTH-type transcriptional repressor NanR | 4.35 | 6.49E-03 |
| Inositol 2-dehydrogenase/D-chiro-inositol 3-dehydrogenase | 4.35 | 6.49E-03 |
| IS1380 family transposase ISMav8 | 4.35 | 6.49E-03 |
| IS256 family transposase ISArsp4 | 4.35 | 6.49E-03 |
| Lipoprotein LprN | 4.35 | 6.49E-03 |
| Long-chain-fatty-acid--CoA ligase | 4.35 | 6.49E-03 |
| Long-chain-fatty-acid--CoA ligase FadD13 | 4.35 | 6.49E-03 |
| Medium-chain fatty-acid--CoA ligase | 4.35 | 6.49E-03 |
| Methyl-branched lipid omega-hydroxylase | 4.35 | 6.49E-03 |
| Mitomycin radical oxidase | 4.35 | 6.49E-03 |
| Modification methylase PvuII | 4.35 | 6.49E-03 |
| NAD/NADP-dependent betaine aldehyde dehydrogenase | 4.35 | 6.49E-03 |
| Nucleoside triphosphatase NudI | 4.35 | 6.49E-03 |
| p-cumate 2,3-dioxygenase system, large oxygenase component | 4.35 | 6.49E-03 |
| putative 3-ketosteroid-9-alpha-monooxygenase, oxygenase component | 4.35 | 6.49E-03 |
| Putative 3-oxopropanoate dehydrogenase | 4.35 | 6.49E-03 |
| Putative acyl-CoA dehydrogenase FadE17 | 4.35 | 6.49E-03 |
| Putative aminoglycoside phosphotransferase | 4.35 | 6.49E-03 |
| putative CoA-transferase | 4.35 | 6.49E-03 |
| Putative coenzyme F420-dependent oxidoreductase | 4.35 | 6.49E-03 |
| Putative cytochrome P450 143 | 4.35 | 6.49E-03 |
| Putative diacyglycerol O-acyltransferase | 4.35 | 6.49E-03 |
| putative enoyl-CoA hydratase 1 | 4.35 | 6.49E-03 |
| Putative enoyl-CoA hydratase EchA13 | 4.35 | 6.49E-03 |
| putative enoyl-CoA hydratase echA8 | 4.35 | 6.49E-03 |
| putative HTH-type transcriptional regulator | 4.35 | 6.49E-03 |
| putative MFS-type transporter EfpA | 4.35 | 6.49E-03 |
| Putative NAD(P)H nitroreductase YodC | 4.35 | 6.49E-03 |
| putative oxidoreductase | 4.35 | 6.49E-03 |
| Putative oxidoreductase SadH | 4.35 | 6.49E-03 |
| putative PPE family protein PPE32 | 4.35 | 6.49E-03 |
| Putative S-adenosyl-L-methionine-dependent methyltransferase | 4.35 | 6.49E-03 |
| Putative short-chain type dehydrogenase/reductase | 4.35 | 6.49E-03 |
| Pyruvate, phosphate dikinase | 4.35 | 6.49E-03 |
| Quinol oxidase subunit 3 | 4.35 | 6.49E-03 |
| Restriction enzyme BgcI subunit alpha | 4.35 | 6.49E-03 |
| Restriction enzyme BgcI subunit beta | 4.35 | 6.49E-03 |
| Short-chain-enoyl-CoA hydratase | 4.35 | 6.49E-03 |
| Siderophore export accessory protein MmpS5 | 4.35 | 6.49E-03 |
| Siderophore exporter MmpL4 | 4.35 | 6.49E-03 |
| Succinyl-CoA--L-malate CoA-transferase alpha subunit | 4.35 | 6.49E-03 |
| Thioredoxin-like reductase | 4.35 | 6.49E-03 |
| Toxin RelG | 4.35 | 6.49E-03 |
| tRNA-Arg(tcg) | 4.35 | 6.49E-03 |
| Tyrosine recombinase XerC | 4.35 | 6.49E-03 |
| UvrABC system protein B | 4.35 | 6.49E-03 |
| Formate hydrogenlyase subunit 4 | 4.35 | 1.88E-02 |
| Formate hydrogenlyase subunit 7 | 4.35 | 1.88E-02 |
| Hydrogenase-4 component B | 4.35 | 1.88E-02 |
| Hydrogenase-4 component E | 4.35 | 1.88E-02 |
| Hydrogenase-4 component G | 4.35 | 1.88E-02 |
| NAD(P)H-quinone oxidoreductase subunit 2, chloroplastic | 4.35 | 1.88E-02 |
| Bile acid-coenzyme A ligase | 3.77 | 4.54E-04 |
| Caffeyl-CoA reductase-Etf complex subunit CarC | 3.77 | 4.54E-04 |
| IS1380 family transposase ISMav7 | 3.77 | 4.54E-04 |
| Putative ketoacyl reductase | 3.77 | 4.54E-04 |
| Putative short-chain type dehydrogenase/reductase/MSMEI_5872 | 3.77 | 4.54E-04 |
| Transcriptional regulator NanR | 3.77 | 4.54E-04 |
| Acyl-CoA thioesterase 2 | 3.35 | 3.61E-02 |
| IS3 family transposase IS987 | 3.35 | 6.61E-03 |
| Epoxyqueuosine reductase | 2.94 | 1.12E-02 |
| IS256 family transposase IS1512 | 2.94 | 1.12E-02 |
| DNA replication and repair protein RecF | 2.61 | 1.74E-02 |
| Insertion element IS6110 uncharacterized 12.0 kDa protein | 2.61 | 1.74E-02 |
| IS110 family transposase IS1547 | 2.35 | 2.54E-02 |
| IS3 family transposase IS3501 | 2.35 | 2.54E-02 |
| Maltokinase | 2.21 | 7.86E-04 |
| IS256 family transposase IS666 | 2.21 | 1.14E-03 |
| Coenzyme A biosynthesis bifunctional protein CoaBC | 1.67 | 5.33E-03 |
| tRNA-Thr(tgt) | 1.67 | 5.33E-03 |
| Arsenate-mycothiol transferase ArsC1 | 1.58 | 6.89E-03 |
| Arsenical-resistance protein Acr3 | 1.58 | 6.89E-03 |
| Cadmium-induced protein CadI | 1.58 | 6.89E-03 |
| DNA-invertase hin | 1.58 | 6.89E-03 |
| Protein ArsC | 1.58 | 6.89E-03 |
| Putative prophage phiRv2 integrase | 1.58 | 6.89E-03 |
| PE family immunomodulator PE5 | 1.28 | 1.39E-02 |
| IS256 family transposase ISMysp7 | 1.28 | 1.72E-02 |
| Putative cytochrome P450 123 | -0.68 | 3.61E-02 |
| NADH pyrophosphatase | -0.74 | 6.49E-03 |
| IS1182 family transposase ISMgi2 | -1.65 | 1.11E-02 |
| putative PPE family protein PPE3 | -1.65 | 1.11E-02 |
| Glutathionyl-hydroquinone reductase YqjG | -1.78 | 5.33E-03 |
| ECF RNA polymerase sigma-E factor | -1.90 | 2.24E-03 |
| putative arabinosyltransferase A | -1.90 | 2.24E-03 |
| Surfactin synthase subunit 2 | -1.90 | 2.59E-02 |
| Aklaviketone reductase DauE | -1.93 | 1.62E-03 |
| Aliphatic sulfonates import ATP-binding protein SsuB | -1.93 | 1.62E-03 |
| Disulfide bond formation protein D | -1.93 | 1.62E-03 |
| Fatty acid oxidation complex subunit alpha | -1.93 | 1.62E-03 |
| Haloalkane dehalogenase 1 | -1.93 | 1.62E-03 |
| Inner membrane protein RclC | -1.93 | 1.62E-03 |
| Peroxiredoxin | -1.93 | 1.62E-03 |
| Putative aliphatic sulfonates transport permease protein SsuC | -1.93 | 1.62E-03 |
| Putative aliphatic sulfonates-binding protein | -1.93 | 1.62E-03 |
| putative ribonucleotide transport ATP-binding protein mkl | -1.93 | 1.62E-03 |
| Copper chaperone CopZ | -2.01 | 1.15E-02 |
| Copper-exporting P-type ATPase | -2.01 | 1.15E-02 |
| Copper-sensing transcriptional repressor RicR | -2.01 | 1.15E-02 |
| Dimodular nonribosomal peptide synthase | -2.01 | 1.15E-02 |
| N(6)-hydroxylysine O-acetyltransferase | -2.01 | 1.15E-02 |
| NAD(P)H-quinone oxidoreductase subunit 1, chloroplastic | -2.01 | 1.15E-02 |
| NAD(P)H-quinone oxidoreductase subunit 4L, chloroplastic | -2.01 | 1.15E-02 |
| NADH-quinone oxidoreductase subunit 12 | -2.01 | 1.15E-02 |
| NADH-quinone oxidoreductase subunit M | -2.01 | 1.15E-02 |
| Narbonolide/10-deoxymethynolide synthase PikA3, module 5 | -2.01 | 1.15E-02 |
| Phenyloxazoline synthase MbtB | -2.01 | 1.15E-02 |
| putative copper-exporting P-type ATPase V | -2.01 | 1.15E-02 |
| Putative inactive phenolphthiocerol synthesis polyketide synthase type I Pks15 | -2.01 | 1.15E-02 |
| Ubiquinone/menaquinone biosynthesis C-methyltransferase UbiE | -2.04 | 5.24E-04 |
| Alanine dehydrogenase | -2.04 | 8.39E-03 |
| Diaminopimelate decarboxylase | -2.04 | 8.39E-03 |
| ESAT-6-like protein EsxN | -2.04 | 8.39E-03 |
| Multidrug resistance protein MdtH | -2.04 | 8.39E-03 |
| Multidrug resistance protein MdtK | -2.04 | 8.39E-03 |
| Sugar-phosphatase AraL | -2.04 | 8.39E-03 |
| Divalent metal cation transporter MntH | -2.08 | 5.91E-03 |
| Multicopper oxidase MmcO | -2.08 | 5.91E-03 |
| N-ethylmaleimide reductase | -2.08 | 5.91E-03 |
| putative manganese/zinc-exporting P-type ATPase | -2.08 | 5.91E-03 |
| putative PPE family protein PPE29 | -2.08 | 5.91E-03 |
| RNA pyrophosphohydrolase | -2.11 | 2.07E-04 |
| Cadmium, cobalt and zinc/H( )-K( ) antiporter | -2.11 | 4.01E-03 |
| ESAT-6-like protein EsxP | -2.11 | 4.01E-03 |
| putative cation-transporting ATPase G | -2.11 | 4.01E-03 |
| Transcriptional regulator BlaI | -2.11 | 4.01E-03 |
| IS481 family transposase ISMav5 | -2.14 | 2.59E-03 |
| Acrylyl-CoA reductase AcuI | -2.17 | 1.58E-03 |
| Peroxyureidoacrylate/ureidoacrylate amidohydrolase RutB | -2.17 | 1.58E-03 |
| Transcriptional regulator AcuR | -2.17 | 1.58E-03 |
| D-inositol-3-phosphate glycosyltransferase | -2.20 | 8.94E-04 |
| dTDP-4-dehydrorhamnose 3,5-epimerase | -2.20 | 8.94E-04 |
| Putative UDP-kanosamine synthase oxidoreductase subunit | -2.20 | 8.94E-04 |
| UDP-N-acetylglucosamine--N-acetylmuramyl-(pentapeptide) pyrophosphoryl-undecaprenol N-acetylglucosamine transferase | -2.20 | 8.94E-04 |
| Pentalenene oxygenase | -2.26 | 1.98E-04 |
| tRNA-Ser(gct) | -2.32 | 1.36E-05 |
