# Supplementary material for: Comparative Genomics of Mycobacterium avium Complex Reveals Signatures of Environment-Specific Adaptation and Community Acquisition
Source: mSystems. 2021 Oct 19;6(5):e01194-21. doi: 10.1128/mSystems.01194-21 (PMC8525567; doi:10.1128/mSystems.01194-21)
Supplement: TABLE S4 [file msystems.01194-21-st004.docx]

**Supplemental Table 4**

| **Gene** | **Log(2) fold change** | **P-value** |
| --- | --- | --- |
| IS3 family transposase ISMyma3 | 6.35 | 3.03E-06 |
| ECF RNA polymerase sigma-E factor | 5.26 | 7.12E-06 |
| Inner membrane protein RclC | 4.94 | 4.07E-13 |
| Disulfide bond formation protein D | 4.30 | 8.12E-13 |
| Haloalkane dehalogenase 1 | 4.30 | 8.12E-13 |
| Putative metal-dependent hydrolase YcfH | 4.14 | 6.42E-05 |
| Lipoprotein LpqS | 4.14 | 4.20E-07 |
| Acrylyl-CoA reductase AcuI | 4.00 | 9.88E-16 |
| Transcriptional regulator AcuR | 3.97 | 9.88E-16 |
| N-ethylmaleimide reductase | 3.94 | 4.76E-14 |
| HTH-type transcriptional regulator CmtR | 3.88 | 2.65E-09 |
| Sugar-phosphatase AraL | 3.75 | 1.64E-13 |
| Voltage-gated ClC-type chloride channel ClcB | 3.73 | 3.41E-07 |
| Zinc transporter ZitB | 3.73 | 3.41E-07 |
| Transcriptional regulatory protein SrrA | 3.62 | 2.64E-05 |
| S-sulfocysteine synthase | 3.62 | 1.79E-08 |
| Putative cation-transporting ATPase G | 3.59 | 7.60E-14 |
| Multidrug resistance protein MdtH | 3.56 | 3.15E-13 |
| Surfactin synthase subunit 2 | 3.50 | 1.36E-10 |
| Multidrug resistance protein MdtK | 3.47 | 8.13E-13 |
| Copper chaperone CopZ | 3.46 | 2.65E-12 |
| L-glutamine:2-deoxy-scyllo-inosose aminotransferase | 3.43 | 2.64E-05 |
| NAD(P)F-quinone oxidoreductase subunit 4L, chloroplastic | 3.40 | 2.65E-12 |
| Transcriptional regulatory protein WalR | 3.37 | 5.97E-05 |
| N(6)-hydroxylysine O-acetyltransferase | 3.35 | 2.65E-12 |
| NADH-quinone oxidoreductase subunit 12 | 3.30 | 2.65E-12 |
| NAD(P)F-quinone oxidoreductase subunit 1, chloroplastic | 2.97 | 2.65E-12 |
| Putative copper-exporting P-type ATPase V | 2.95 | 2.65E-12 |
| Arsenite methyltransferase | 2.86 | 6.65E-09 |
| Glutaredoxin arsenate reductase | 2.86 | 6.65E-09 |
| Insertion sequence IS5376 ATP-binding protein | 2.72 | 1.05E-06 |
| Putative aldehyde dehydrogenase AldA | 2.62 | 8.70E-10 |
| RNA pyrophosphohydrolase | 2.61 | 2.47E-15 |
| Peroxiredoxin | 2.49 | 8.12E-13 |
| Putative inactive phenolphthiocerol synthesis polyketide synthase type I Pks15 | 2.09 | 2.65E-12 |
| ATP-dependent RecD-like DNA helicase | 1.94 | 6.42E-05 |
| Putative manganese catalase | 1.92 | 3.41E-07 |
| IS256 family transposase IS1512 | 1.68 | 1.29E-09 |
| Haloalkane dehalogenase 2 | 1.68 | 8.12E-13 |
| NAD(P)F-quinone oxidoreductase subunit 2, chloroplastic | 1.63 | 2.65E-12 |
| Doxorubicin resistance ABC transporter permease protein DrrB | 1.51 | 5.85E-12 |
| NAD(P)F-quinone oxidoreductase subunit 3, chloroplastic | 1.35 | 1.15E-05 |
| Arsenical-resistance protein Acr3 | 1.34 | 6.65E-09 |
| Biphenyl-2,3-diol 1,2-dioxygenase | 1.30 | 3.07E-08 |
| IS21 family transposase ISMyma9 | 1.19 | 2.11E-06 |
| IS1634 family transposase IS1549 | 1.06 | 8.49E-07 |
| HTH-type transcriptional regulator MmpR5 | 1.00 | 2.24E-07 |
| Hydroperoxy fatty acid reductase gpx2 | 0.92 | 6.31E-06 |
| Alpha-acetolactate decarboxylase | 0.91 | 6.15E-10 |
| Putative PPE family protein PPE29 | 0.91 | 3.15E-13 |
| Neutral endopeptidase | 0.90 | 2.86E-08 |
| Anti-sigma-L factor RslA | 0.90 | 7.81E-07 |
| Copper-exporting P-type ATPase | 0.87 | 2.65E-12 |
| NADH-quinone oxidoreductase subunit M | 0.84 | 2.65E-12 |
| Aklaviketone reductase DauE | 0.84 | 8.12E-13 |
| 8-demethyl-8-(2,3-dimethoxy-alpha-L-rhamnosyl)-tetracenomycin-C 4'-O-methyltransferase | 0.82 | 1.99E-07 |
| Transcriptional regulator BlaI | 0.81 | 7.60E-14 |
| Tyrosine recombinase XerC | 0.80 | 7.60E-14 |
| Mercuric resistance operon regulatory protein | 0.78 | 2.65E-09 |
| Glucose--fructose oxidoreductase | 0.77 | 4.88E-07 |
| Diacylglycerol kinase | 0.76 | 4.37E-07 |
| Phospho-2-dehydro-3-deoxyheptonate aldolase, Phe-sensitive | 0.76 | 6.65E-09 |
| Putative arabinosyltransferase A | 0.76 | 2.65E-12 |
| Putative cation efflux system protein | 0.74 | 4.63E-05 |
| Alanine dehydrogenase | 0.73 | 8.13E-13 |
| Dihydrolipoyl dehydrogenase | 0.69 | 2.85E-05 |
| Sulfoxide reductase catalytic subunit YedY | 0.68 | 2.85E-05 |
| Glutathionyl-hydroquinone reductase YqjG | 0.68 | 7.27E-11 |
| PE-PGRS family protein PE PGRS16 | 0.66 | 7.81E-07 |
| 3-beta-hydroxycholanate 3-dehydrogenase | 0.64 | 4.37E-07 |
| Putative aminoacrylate hydrolase RutD | 0.64 | 4.37E-07 |
| Phenyloxazoline synthase MbtB | 0.61 | 2.65E-12 |
| Thioredoxin-like reductase | 0.60 | 5.77E-08 |
| IS3 family transposase ISMysp3 | 0.59 | 1.93E-06 |
| Copper-sensing transcriptional repressor RicR | 0.56 | 2.65E-12 |
| ESAT-6-like protein EsxN | 0.53 | 2.08E-07 |
| Putative ribonucleotide transport ATP-binding protein mkl | 0.51 | 8.12E-13 |
| Bifunctional oligoribonuclease and PAP phosphatase NrnA | 0.48 | 2.64E-05 |
| Threonine synthase | 0.48 | 2.64E-05 |
| Aliphatic sulfonates import ATP-binding protein SsuB | 0.47 | 8.12E-13 |
| Haloalkane dehalogenase | 0.46 | 2.85E-05 |
| Putative aliphatic sulfonates transport permease protein SsuC | 0.46 | 8.12E-13 |
| dTDP-L-rhamnose 4-epimerase | 0.46 | 1.99E-07 |
| Putative aliphatic sulfonates-binding protein | 0.45 | 8.12E-13 |
| Multicopper oxidase MmcO | 0.44 | 3.15E-13 |
| Transcription elongation factor GreA | 0.43 | 6.65E-09 |
| 50S ribosomal protein L28 | 0.42 | 3.61E-05 |
| Peptidoglycan endopeptidase RipB | 0.41 | 6.42E-05 |
| NDMA-dependent alcohol dehydrogenase | 0.40 | 4.37E-07 |
| Putative cytochrome P450 138 | 0.40 | 3.41E-07 |
| IS110 family transposase IS1110 | 0.38 | 2.86E-08 |
| ESAT-6-like protein EsxP | 0.36 | 7.60E-14 |
| Iron-dependent extradiol dioxygenase | 0.35 | 6.31E-06 |
| Diaminopimelate decarboxylase | 0.35 | 8.13E-13 |
| ATP-dependent zinc metalloprotease FtsH | 0.34 | 8.28E-05 |
| Alkanesulfonate monooxygenase | 0.33 | 8.12E-13 |
| Baeyer-Villiger monooxygenase | 0.31 | 4.37E-07 |
| Putative PPE family protein PPE32 | 0.31 | 2.65E-12 |
| Protein MbtH | 0.31 | 3.03E-05 |
| Putative monooxygenase | 0.30 | 4.88E-07 |
| DNA protection during starvation protein | 0.30 | 3.41E-07 |
| Putative aldehyde dehydrogenase | 0.29 | 2.86E-08 |
| Succinate-semialdehyde dehydrogenase | 0.28 | 2.85E-05 |
| Divalent metal cation transporter MntH | 0.27 | 3.15E-13 |
| Adaptive-response sensory-kinase SasA | 0.27 | 2.64E-05 |
| 18 kDa heat shock protein | 0.27 | 6.65E-09 |
| Fatty acid oxidation complex subunit alpha | 0.26 | 8.12E-13 |
| (R)-benzylsuccinyl-CoA dehydrogenase | 0.26 | 3.41E-07 |
| Epoxide hydrolase A | 0.25 | 3.41E-07 |
| Putative coenzyme F420-dependent oxidoreductase | 0.25 | 3.41E-07 |
| Anti-sigma-F factor antagonist RsfB | 0.24 | 2.85E-05 |
| 4,5:9,10-diseco-3-hydroxy-5,9,17-trioxoandrosta-1(10),2-diene-4-oate hydrolase | 0.24 | 3.07E-08 |
| Lipoprotein LprN | 0.23 | 2.25E-11 |
| Linear gramicidin synthase subunit D | 0.23 | 2.86E-08 |
| Alcohol dehydrogenase | 0.22 | 3.41E-07 |
| Immunogenic protein MPB64 | 0.22 | 6.31E-06 |
| Putative glycosyltransferase | 0.22 | 2.86E-08 |
| 3-oxoacyl-[acyl-carrier-protein] reductase FabG | 0.20 | 8.12E-06 |
| Putative membrane protein | 0.19 | 6.65E-09 |
| Putative PPE family protein PPE3 | 0.19 | 2.86E-08 |
| Putative phospholipid ABC transporter permease protein MlaE | 0.17 | 4.37E-07 |
| Cytochrome P450 130 | 0.17 | 4.88E-07 |
| DNA ligase | 0.16 | 2.85E-05 |
| Siderophore export accessory protein MmpS5 | 0.16 | 2.86E-08 |
| 1,4-dihydroxy-2-naphthoyl-CoA synthase | 0.16 | 4.88E-07 |
| Vitamin B12 import ATP-binding protein BtuD | 0.14 | 2.86E-08 |
| Putative cytochrome P450 123 | 0.14 | 9.19E-10 |
| Putative S-adenosyl-L-methionine-dependent methyltransferase | 0.10 | 2.86E-08 |
| Succinyl-CoA--L-malate CoA-transferase beta subunit | 0.09 | 1.79E-08 |
| Siderophore exporter MmpL4 | 0.08 | 2.86E-08 |
| Tyrosine recombinase XerD | 0.08 | 2.86E-08 |
| F420-dependent hydroxymycolic acid dehydrogenase | 0.08 | 3.41E-07 |
| ESX-3 secretion system ATPase EccB3 | 0.07 | 2.86E-08 |
| HTH-type transcriptional repressor YvoA | 0.07 | 1.52E-05 |
| Acyltransferase papA3 | 0.07 | 6.65E-09 |
| Putative HTH-type transcriptional regulator | 0.07 | 3.41E-07 |
| Putative prophage phiRv2 integrase | 0.07 | 1.52E-05 |
| Arylsulfatase | 0.06 | 4.88E-07 |
| HTH-type transcriptional repressor FabR | 0.05 | 3.41E-07 |
| Maltokinase | 0.05 | 8.12E-13 |
| NAD(P) transhydrogenase subunit alpha part 1 | 0.05 | 7.81E-07 |
| Putative two-component membrane permease complex subunit SMU 747c | 0.05 | 2.86E-08 |
| NADPH oxidoreductase | 0.04 | 3.41E-07 |
| Putative oxidoreductase | 0.03 | 4.37E-07 |
| Crotonobetainyl-CoA dehydrogenase | 0.00 | 3.41E-07 |
| Acyl-CoA dehydrogenase FadE34 | -0.03 | 2.86E-08 |
| Medium-chain fatty-acid--CoA ligase | -0.03 | 2.86E-08 |
| GDP-mannose 4,6-dehydratase | -0.04 | 7.02E-11 |
| Dimodular nonribosomal peptide synthase | -0.08 | 2.65E-12 |
| Deazaflavin-dependent nitroreductase | -0.09 | 2.86E-08 |
| Putative oxidoreductase EphD | -0.10 | 1.15E-05 |
| D-alanine--poly(phosphoribitol) ligase subunit 1 | -0.15 | 2.86E-08 |
| Aurachin B dehydrogenase | -0.17 | 3.41E-07 |
| Methyl-branched lipid omega-hydroxylase | -0.22 | 2.86E-08 |
| Putative enoyl-CoA hydratase 1 | -0.22 | 5.77E-08 |
| PPE family protein PPE4 | -0.24 | 2.86E-08 |
| Transcriptional regulator LytR | -0.24 | 3.41E-07 |
| (-)-trans-carveol dehydrogenase | -0.27 | 2.86E-08 |
| PE family immunomodulator PE5 | -0.35 | 4.23E-06 |
| NADPH-dependent stearoyl-CoA 9-desaturase | -0.38 | 2.65E-12 |
| Putative doxorubicin resistance ABC transporter permease protein DrrC | -0.43 | 2.01E-11 |
| Long-chain-fatty-acid--AMP ligase FadD28 | -0.45 | 2.86E-08 |
| NADH-quinone oxidoreductase subunit I | -0.47 | 2.65E-12 |
| 30S ribosomal protein S14 | -0.48 | 2.86E-08 |
| 30S ribosomal protein S18 | -0.48 | 2.86E-08 |
| Zinc uptake regulation protein | -0.48 | 2.86E-08 |
| Sulfolipid-1 exporter Sap | -0.50 | 2.86E-08 |
| GDP-L-fucose synthase | -0.63 | 2.01E-11 |
| Putative glutamate--cysteine ligase 2 | -0.64 | 2.65E-12 |
| IS110 family transposase IS1547 | -0.73 | 3.03E-05 |
| Putative antitoxin VapB5 | -0.78 | 5.34E-05 |
| Non-haem bromoperoxidase BPO-A2 | -0.87 | 5.77E-08 |
| Ribosome-associated ATPase | -0.90 | 5.77E-08 |
| Glucose 1-dehydrogenase | -0.98 | 2.86E-08 |
| Biotin biosynthesis cytochrome P450 | -1.13 | 2.86E-08 |
| Multidrug resistance protein Stp | -1.83 | 2.86E-08 |
| IS3 family transposase ISMyma10 | -2.03 | 2.86E-08 |
| Multifunctional dye peroxidase DyP2 | -2.30 | 1.27E-05 |
| IS110 family transposase ISMpa1 | -3.25 | 7.34E-08 |
| Esterase FrsA | -4.49 | 2.86E-08 |
| Putative PPE family protein PPE37 | -4.49 | 2.86E-08 |
| 50S ribosomal protein L31 type B | -4.67 | 2.86E-08 |
| Alpha-ketoglutarate-dependent taurine dioxygenase | -4.67 | 2.86E-08 |
| Dicitrate transport ATP-binding protein FecE | -4.67 | 2.86E-08 |
| Putative ABC transporter permease protein | -4.67 | 2.86E-08 |
| Thioesterase PikA5 | -4.67 | 2.86E-08 |
| Putative metal chaperone YciC | -4.73 | 2.86E-08 |
| Energy-coupling factor transporter transmembrane protein EcfT | -4.83 | 2.86E-08 |
| Tetracycline repressor protein class D | -5.03 | 2.65E-12 |
| IS30 family transposase ISBlo4 | -5.24 | 7.34E-08 |
| IS481 family transposase ISMav4 | -5.86 | 1.80E-05 |
