# Supplementary material for: Comparative Genomics of Mycobacterium avium Complex Reveals Signatures of Environment-Specific Adaptation and Community Acquisition
Source: mSystems. 2021 Oct 19;6(5):e01194-21. doi: 10.1128/mSystems.01194-21 (PMC8525567; doi:10.1128/mSystems.01194-21)
Supplement: TABLE S5 [file msystems.01194-21-st005.docx]

**Supplemental Table 5**

| **Gene** | **Enrichment** | **P-value** |
| --- | --- | --- |
| RNA polymerase sigma factor RpoD | Enriched in *M. avium* | 5.22E-44 |
| Ribonuclease BN | Enriched in *M. avium* | 5.41E-41 |
| Acyl-CoA transferase domain of IgrD / Nucleic-acid-binding domain of IgrD | Enriched in *M. avium* | 3.61E-41 |
| 3-hydroxyacyl-CoA dehydrogenase | Enriched in *M. avium* | 1.74E-38 |
| MCE-family protein MceC | Enriched in *M. avium* | 6.36E-35 |
| MCE-family protein MceF | Enriched in *M. avium* | 2.74E-34 |
| Phosphoserine phosphatase (EC 3.1.3.3) / Acyl-CoA:1-acyl-sn-glycerol-3-phosphate acyltransferase | Enriched in *M. avium* | 2.04E-33 |
| Probable short-chain type dehydrogenase/reductase (EC 1.-.-.-) | Enriched in *M. avium* | 4.72E-31 |
| Polyphosphate kinase 2 (EC 2.7.4.1) | Enriched in *M. avium* | 2.13E-30 |
| 2-methylfumaryl-CoA hydratase (EC 4.2.1.148) | Enriched in *M. avium* | 4.32E-29 |
| Putative transmembrane transport protein | Enriched in *M. avium* | 1.30E-27 |
| Metallopeptidase | Enriched in *M. avium* | 4.99E-26 |
| Possible amide hydrolase Rv1333 | Enriched in *M. avium* | 1.63E-22 |
| Thiamin-phosphate pyrophosphorylase (EC 2.5.1.3) | Enriched in *M. avium* | 5.82E-13 |
| Two component transcriptional regulatory protein DevR | Enriched in *M. avium* | 6.39E-11 |
| Steroid C27-monooxygenase (EC 1.14.13.141) | Enriched in *M. avium* | 3.27E-08 |
| Low molecular weight protein tyrosine phosphatase (EC 3.1.3.48) | Enriched in *M. avium* | 9.87E-08 |
| RNA polymerase ECF-type sigma factor | Enriched in *M. avium* | 9.71E-07 |
| Multidrug efflux pump P55 | Enriched in *M. avium* | 9.65E-06 |
| Integral membrane protein EccD3, component of Type VII secretion system ESX-3 | Enriched in *M. avium* | 1.15E-05 |
| Pantoate--beta-alanine ligase (EC 6.3.2.1) | Enriched in *M. avium* | 1.68E-05 |
| WhiB family transcriptional regulator | Enriched in *M. avium* | 0.00013 |
| Iron-regulated heparin binding hemagglutinin HbhA (Adhesin) | Enriched in *M. avium* | 0.00022 |
| Lysine N-acyltransferase MbtK (EC 2.3.1.-) @ Siderophore synthetase small component, acetyltransferase | Enriched in *M. avium* | 0.00048 |
| Efflux transmembrane permease Rv1747 family | Enriched in *M. avium* | 0.00618 |
| 3-oxoacyl-[acyl-carrier-protein] synthase, KASII (EC 2.3.1.179) | Enriched in *M. avium* | 0.00968 |
| NADH-ubiquinone oxidoreductase chain G (EC 1.6.5.3) | Enriched in *M. avium* | 0.03800 |
| Dipeptide ABC transporter, ATP-binding protein DppD (TC 3.A.1.5.2) | Depleted in *M. avium* | 4.43E-36 |
| Acyl carrier protein | Depleted in *M. avium* | 3.16E-33 |
| Efflux ABC transporter, permease/ATP-binding protein SCO2464 | Depleted in *M. avium* | 5.05E-33 |
| Phosphate ABC transporter, substrate-binding protein PstS (TC 3.A.1.7.1) | Depleted in *M. avium* | 1.97E-30 |
| Cytochrome c oxidase polypeptide II (EC 1.9.3.1) | Depleted in *M. avium* | 1.85E-27 |
| Ribonucleotide reductase of class Ib (aerobic), beta subunit (EC 1.17.4.1) | Depleted in *M. avium* | 8.59E-26 |
| Bifunctional salicyl-AMP ligase/salicyl-S-MbtB synthetase MbtA @ adenylation component of NRPS | Depleted in *M. avium* | 4.34E-19 |
| Acyl-CoA dehydrogenase (EC 1.3.8.1), Mycobacterial subgroup FadE32 | Depleted in *M. avium* | 6.24E-15 |
| Nucleoside triphosphate pyrophosphohydrolase MazG (EC 3.6.1.8) | Depleted in *M. avium* | 1.04E-13 |
| Methoxy mycolic acid synthase MmaA2 (EC 2.1.1.79) | Depleted in *M. avium* | 8.07E-10 |
| PPE family protein, PPW subgroup => PPE2 | Depleted in *M. avium* | 3.14E-08 |
| Segregation and condensation protein B | Depleted in *M. avium* | 6.68E-08 |
| Mobile element protein | Depleted in *M. avium* | 1.20E-07 |
| Cholesterol oxidase (EC 1.1.3.6) @ Steroid Delta(5)->Delta(4)-isomerase (EC 5.3.3.1) | Depleted in *M. avium* | 5.52E-07 |
| Cysteine synthesis adenylyltransferase/sulfurtransferase | Depleted in *M. avium* | 0.00033 |
| Polyphosphate glucokinase (EC 2.7.1.63) | Depleted in *M. avium* | 0.00042 |
| HNH endonuclease domain protein | Depleted in *M. avium* | 0.00050 |
| Iron siderophore ABC transporter, permease/ATP-binding protein IrtA | Depleted in *M. avium* | 0.00431 |
| L-asparagine permease | Depleted in *M. avium* | 0.01623 |
| Hydrolase, haloacid dehalogenase-like family | Depleted in *M. avium* | 0.01588 |
